# The Estrogen–Autophagy Axis: Insights into Cytoprotection and Therapeutic Potential in Cancer and Infection

**DOI:** 10.3390/ijms252312576

**Published:** 2024-11-22

**Authors:** Ying Zhao, Daniel J. Klionsky, Xin Wang, Qiaoying Huang, Zixin Deng, Jin Xiang

**Affiliations:** 1 Key Laboratory of Combinatorial Biosynthesis and Drug Discovery, Ministry of Education, School of Pharmaceutical Sciences, Wuhan University, Wuhan 430071, China; zying1305@163.com (Y.Z.); w919074903@163.com (X.W.); 2021313061063@whu.edu.cn (Q.H.); zxdeng@sjtu.edu.cn (Z.D.); 2 Life Sciences Institute, University of Michigan, Mary Sue Coleman Hall, 210 Washtenaw Avenue, Ann Arbor, MI 48109-2216, USA; klionsky@umich.edu

**Keywords:** autophagy, cancer, estrogen, immunity, infection

## Abstract

Macroautophagy, commonly referred to as autophagy, is an essential cytoprotective mechanism that plays a significant role in cellular homeostasis. It has emerged as a promising target for drug development aimed at treating various cancers and infectious diseases. However, the scientific community has yet to reach a consensus on the most effective approach to manipulating autophagy, with ongoing debates about whether its inhibition or stimulation is preferable for managing these complex conditions. One critical factor contributing to the variability in treatment responses for both cancers and infectious diseases is estrogen, a hormone known for its diverse biological effects. Given the strong correlations observed between estrogen signaling and autophagy, this review seeks to summarize the intricate molecular mechanisms that underlie the dual cytoprotective effects of estrogen signaling in conjunction with autophagy. We highlight recent findings from studies that involve various ligands, disease contexts, and cell types, including immune cells. Furthermore, we discuss several factors that regulate autophagy in the context of estrogen’s influence. Ultimately, we propose a hypothetical model to elucidate the regulatory effects of the estrogen–autophagy axis on cell fate. Understanding these interactions is crucial for advancing our knowledge of related diseases and facilitating the development of innovative treatment strategies.

## 1. Introduction

Autophagy is a crucial cytoprotective response that helps cells cope with pathological stresses associated with various diseases, including cancer, ischemia, and infections. It plays a vital role in maintaining the intracellular balance of mass and energy necessary for metabolism, growth, development, and immune function. However, while autophagy is protective in healthy cells, it can also promote drug resistance and nutrient deficiency in cancer cells [1,2]. This dual nature makes autophagy an appealing therapeutic target, as it is intricately linked to a range of metabolic diseases, cancers, developmental disorders, aging, and infectious diseases, including severe acute respiratory syndrome coronavirus 2 (SARS-CoV-2) [3,4].

Estrogen, a key sex hormone, is instrumental in the development of secondary sexual characteristics in females and also exerts various cytoprotective effects in pre-menopausal women [5,6]. The classic estrogen receptors, namely ESR1 (estrogen receptor 1) and ESR2, along with GPER1 (G protein-coupled estrogen receptor 1), play significant regulatory roles in mediating estrogen’s effects [7,8]. A proposed model suggests that 17β-estradiol (E2) modulates autophagy in cancers to help maintain cellular homeostasis [9]. For instance, antagonizing estrogen is often necessary during the treatment of estrogen-related cancers, aligning with the principles of endocrine therapy. However, this model does not fully explain why agonists of ESRs can sometimes provide benefits in cancer treatment [10]. Notably, estrogen to inhibits the proliferation of MDA-MB-231 cells, which are derived from a highly aggressive and poorly differentiated subtype of triple-negative breast cancer [11]. Additionally, clinical trials have indicated that high doses of estrogen may exhibit antitumor effects in postmenopausal breast cancer patients who are resistant to endocrine therapies [12]. Furthermore, some estrogen antagonists have been found to inhibit autophagy [13,14]. Estrogen also plays crucial regulatory roles in the immune response, highlighting the importance of the crosstalk between estrogen signaling and autophagy in the onset and progression of cancer.

This review summarizes the molecular mechanisms underlying the cytoprotective effects resulting from the interplay between estrogen signaling and autophagy. We explore how various ligands and cell types, including immune cells, influence this relationship in the context of disease. Additionally, we discuss factors affecting estrogen regulation of autophagy, such as ESRs, transcription factors, microRNAs, and histone modifications. Finally, we examine the implications of the estrogen–autophagy axis on cell fate to guide future research directions.

## 2. E2 Regulates Autophagy in Diseases

Over the past few decades, there has been a growing body of research exploring the relationship between estrogen signaling and autophagy [15,16]. Most synthetic modulators that influence estrogen’s effects, including ligands for ESRs, were originally developed for the clinical treatment of ESR-positive breast cancers [17]. However, the impact of these estrogen regulators extends beyond cancer, affecting various tissues such as the brain, bone, visceral fat, skin, lymphatic system, adrenal medulla, oral cavity, and eyes. This suggests that the link between estrogen and autophagy may play a role in developing treatments for a range of diseases, not just cancers [14,18,19,20,21,22,23,24,25,26,27,28,29,30,31,32,33,34,35,36,37,38,39,40].

Autophagy is a dynamic process that involves the coordinated action of numerous proteins, which serve as biomarkers, such as MAP1LC3 (microtubule-associated protein 1 light chain 3) and those related to autophagy flux (the MAP1LC3-II:MAP1LC3-I ratio), as well as autophagy substrates including SQSTM1 (sequestosome 1) and the morphological features of autophagosomes and autolysosomes [41]. Additionally, BECN1 (beclin 1) is a crucial regulator of autophagy during the nucleation phase. However, variations in its expression levels appear to correlate more closely with autophagy regulation than with morphological changes or the expression patterns of autophagy biomarkers such as MAP1LC3.

As summarized in Table S1, E2 induces the degradation of ESR1 in MCF-7 cells and increases the number of autophagosomes [18]. Furthermore, E2 upregulate BECN1 via ESR2 in MDA-MB-231 cells [19], promote autophagosome formation and apoptosis in renal cell carcinoma [28], and enhance the expression of MAP1LC3-II in pulmonary artery endothelial cells in a rat model of hypoxia, indicating a protective effect against hypoxia-induced pulmonary hypertension [20,21]. Additionally, E2 stimulates autophagy by upregulating BECN1 and enhancing autophagy flux, which effectively suppresses apoptosis in rat nucleus pulposus cells and osteocytes, as well as human osteoblasts and retinal pigment epithelial cells [22,23,24,26,29]. E2 also increases the ratio of autolysosomes to autophagosomes, demonstrating a significant therapeutic effect in a rat model of Parkinson disease [27]. It also inhibits the expression of certain autophagy-related proteins in response to various conditions, including endometriosis, hypoxia, ischemia, lipopolysaccharides, spinal cord injury, NaAsO_2_ exposure, and ovariectomy [32,36,38,39].

**Figure 1:**
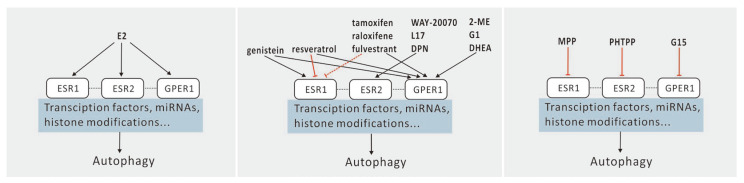
Effects of ESR ligands on autophagy in non-stimulated cells. Dashed lines indicate tissue-specific activation or inhibition. Dotted lines indicate crosstalk between receptors. Sharp arrow (black), activates; blunt arrow (red), inhibits.

Specific modulators have been developed to target the activities of ESR1, ESR2, and GPER1. These include ESR1 antagonists such as tamoxifen, raloxifene, and fulvestrant [17]. Both fulvestrant and resveratrol promote autophagy while limiting the upregulation of autophagy induced by rapamycin or H_2_O_2_ [14,42]. G1, a GPER1 agonist, inhibits excessive autophagy induced by angiotensin II or glutamate [43,44]. Notably, the activities of fulvestrant and G1 are similar to those of E2, as all three act as agonists for GPER1, although some also serve as antagonists for ESR1 (Figure 1). These findings indicate that estrogen regulation has dual effects on autophagy, complicating the determination of whether the type of ligand affects autophagy regulation.

To date, several estrogen antagonists have been synthesized, including the ESR1-selective antagonist 1,3-bis(4-hydroxyphenyl)-4-methyl-5-(4-[2-piperidinylethoxy]phenol)-1H-pyrazole dihydrochloride (MPP), the ESR2-selective antagonist 4-(2-phenyl-5,7-bis[trifluoromethyl]pyrazolo[1,5-a]pyrimidin-3-yl)phenol (PHTPP), and the GPER1 antagonist G15 [45,46]. Both MPP and PHTPP inhibit autophagy by suppressing autophagy flux [46], whereas G15 blocks G1’s action, resulting in the accumulation of MAP1LC3B-II and autophagosomes, similar to the effect of pre-treatment with chloroquine, which increases increase autophagosome accumulation [13,45,47]. Furthermore, silencing ESR1 mRNA using small interfering RNA also inhibits autophagy [43]. Together, these findings suggest that ESR ligands regulate autophagy and that the autophagy induced by estrogen can be blocked with antagonists (Figure 1, Table S2) [13,14,42,43,44,48,49,50,51,52,53,54,55,56,57,58,59,60,61,62,63,64,65,66,67,68,69,70,71].

### 2.1. Expression and Distribution of ESRs Under Normal Physiological and Disease Conditions

**Figure 2:**
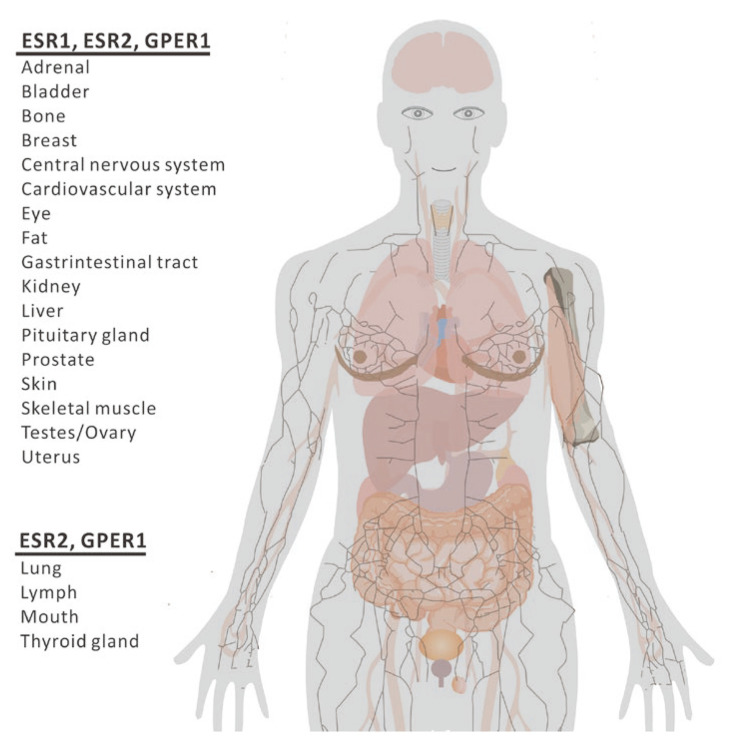
Schematic view of the distribution of ESRs among organs.

ESR1, ESR2, and GPER1 exhibit distinct patterns of distribution and expression in both normal physiological and disease conditions. As illustrated in Figure 2, GPER1 and ESR2 are more broadly distributed among human cell types compared to ESR1. These differences in expression patterns have been identified as potential biomarkers for assessing the risk of malignant cancers, including those of the breast, endometrium, and ovary [72,73,74].

The lymphatic system serves as a common pathway for the spread of breast cancer cells, occurring at a frequency comparable to hematogenous (i.e., blood borne) metastasis. Approximately 75% of primary breast cancers are ESR1-positive, with lymph nodes being the primary metastatic sites for these ESR1-positive breast cancer cells. Notably, the presence of ESR1-positive follicular dendritic cells in lymph nodes has been recognized as a novel prognostic marker for breast cancer, aiding in therapeutic decision-making [63]. For instance, triple-negative breast cancer, which lacks ESR1 expression, has the poorest prognosis among the five molecular subtypes of breast cancer. This is attributed to a high mitotic rate, extensive lymphocyte infiltration, advanced tumor grade, and larger tumor size.

All three estrogen receptors—ESR1, ESR2, and GPER1—are expressed in the pituitary gland [75] and play roles in neuronal-mediated contractions of the ileal tissues in females. In contrast, only ESR1 is found in the ileal tissues of males [76]. This distribution may contribute to the differing prevalence of gastrointestinal cancer and non-erosive reflux disease between genders, with such conditions being more common in men, whereas functional gastrointestinal disorders are frequently observed in women [77,78]. Additionally, all three ESRs have been identified in the human bladder [79]. While both ESR1 and ESR2 (but not GPER1) protect the human bladder and kidney from *Escherichia coli* infections, they also play roles in the carcinogenesis, progression, and chemosensitivity of bladder cancer [80,81]. Furthermore, ESR1-mediated estrogen signaling is crucial for effective liver regeneration following partial hepatectomy [82].

ESR1 significantly contributes to metabolic regulation in skeletal muscle but is not essential for regulating insulin sensitivity or mitochondrial function [83]. In contrast, ESR2 is involved in a sex-specific regulatory mechanism that governs muscle growth and regeneration in female mice [84]. The antifibrogenic effects of estrogen are primarily mediated by ESR2 rather than ESR1 or GPER1 in rats [85]. Notably, ESR2 is the predominant subtype expressed in colon tissues, and E2 may protect against acute colitis through ESR2 activation. An upregulation of ESR2 expression in colon cancer is associated with improved survival outcomes [86].

In the normal thyroid gland, a limited number of follicular cells express ESR1, whereas both follicular and C cells express ESR2, even in the developing fetus [87]. ESR2 is highly expressed in type I and II pneumocytes as well as bronchiolar epithelial cells, whereas ESR1 is undetectable in the adult lung [88]. Furthermore, ESR2 appears to be the predominant estrogen receptor subtype in lung cancer [89], the oral epithelium, and salivary glands [90]. While both ESR1 and ESR2 are present in gastric cancer [86], expression of ESR2 in non-cancerous tissues is significantly higher in female than in male rats. ESR1 is found in only a small percentage of older males with oral squamous cell carcinoma, whereas young females generally do not express ESR1 or the progesterone receptor [91]; however, ESR2 is predominantly expressed in tissue samples from patients with oral cancer [91].

GPER1 plays neuroprotective roles and is associated with vascular pathology in various tissues [92]. This receptor induces pleiotropic effects in metabolically active tissues, including the pancreas, adipose tissue, liver, and skeletal muscle. Research has demonstrated that GPER1 is involved in regulating body weight, feeding behavior, inflammation, and glucose and lipid homeostasis [93]. GPER1 is expressed in the rat pituitary gland, particularly in lactotropic cells [94], and can reduce autophagy in retinal astrocytes under hyperoxic conditions [95]. Moreover, GPER1 expression is upregulated during the differentiation of 3T3-L1 preadipocytes into adipocytes, with higher levels observed in female than male mice [93]. In the liver, GPER1 modulates lipid metabolism, lowers circulating lipid levels, and reduces inflammation, as supported by several preclinical and clinical studies. A hypofunctional genetic variant of GPER1 is linked to elevated plasma low-density lipoprotein cholesterol levels [96].

In uterine circulation, GPER1 triggers a vasoactive response, primarily through the nitric oxide–cyclic guanosine monophosphate signaling pathway in uterine arteries, a mechanism that may be altered during pregnancy [97]. GPER1 is recognized as the primary mediator of estrogen’s effects in the prostate, where its signaling contributes to E2-dependent activation of endothelial nitric oxide production and subsequent vasodilation. It also promotes sodium excretion via an EDN1 (endothelin 1)-dependent pathway in female, but not male, rats, thereby enhancing renal vascular function against ischemia-reperfusion injury. Additionally, GPER1 is present in the skin, which serves as a barrier against microbial pathogens. It is located in the membranes of endothelial cells and various skin cells, including keratinocytes, melanocytes, and dermal fibroblasts [98]. GPER1 activation mitigates pathogenesis in a mouse model of *Staphylococcus aureus* skin infections, reinforcing the role of estrogen in supporting innate immunity against infectious diseases via GPER1.

GPER1 is expressed in various cancers, with higher levels of GPER expression linked to tumor development in some female reproductive system neoplasms and associated with poor prognosis [99]. Furthermore, GPER1 is found in the majority of mantle cell lymphoma cases, suggesting that therapies targeting GPER1 could benefit these patients [99]. Even at low expression levels, GPER1 has been independently associated with lymph node metastasis in papillary thyroid carcinoma [100,101]. Notably, GPER1 expression is higher in lung tumors compared to normal lung tissues [102]. However, it is inversely associated with lymph node metastasis, high-grade tumors, and FN1 (fibronectin 1) expression, while positively correlated with favorable outcomes in patients with triple-negative breast cancer [103]. Downregulation of GPER1 has been linked to poor prognosis in gastric cancer, and it may function as a tumor suppressor by regulating epithelial–mesenchymal transition in this context [104]. GPER1 activation results in the depletion of MYC/c-Myc, inhibition of tumor proliferation, and enhanced immune recognition by tumor cells [105]. Beyond tissue distribution, the intracellular localization of ESRs also varies among different cell types [106,107,108,109] and is closely related to physiological states such as estrogen status. This localization plays a critical role in mediating estrogen signaling and autophagy.

### 2.2. Transcription Factors in Estrogen’s Influence on Autophagy

Autophagy is a dynamic process that involves the coordination of various proteins and can be divided into four key stages: (1) induction, (2) phagophore nucleation and expansion, (3) autophagosome maturation and fusion with endosomes and/or lysosomes, and (4) cargo degradation and recycling. MTOR (mechanistic target of rapamycin kinase) plays a crucial role in regulating autophagy by inhibiting its initiation. When stimulated by estrogen, ESR1 binds to RPTOR/Raptor, a regulatory protein of MTOR complex 1 [110]. This interaction leads to the phosphorylation of Ser104/106 on ESR1, promoting the transcription of target genes associated with ESR1 [110,111].

**Figure 3:**
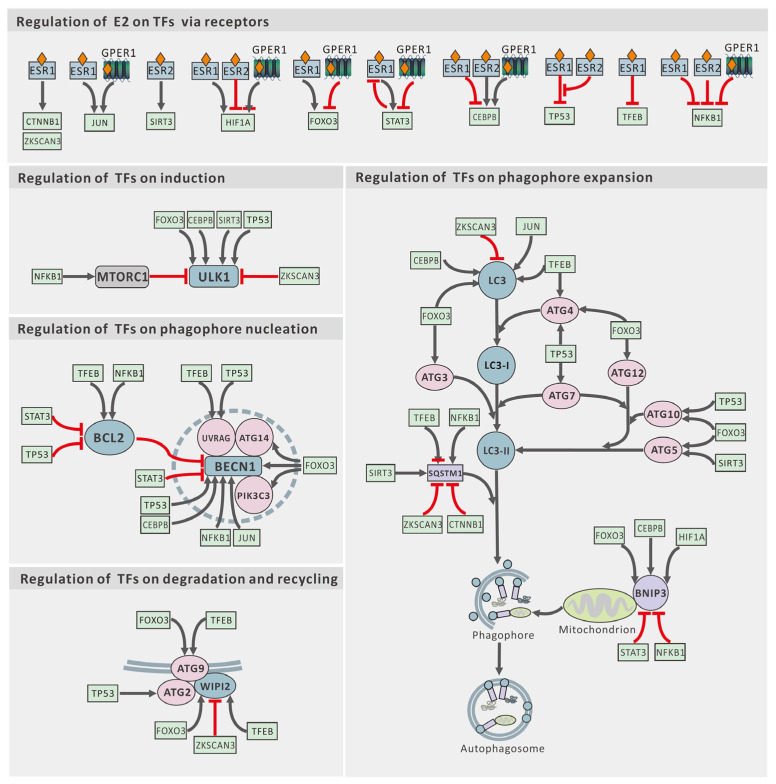
Estrogen regulation of core autophagy proteins via transcription factors. JUN, JUN proto-oncogene, AP-1 transcription factor subunit. Sharp arrow (black), activates; blunt arrow (red), inhibits.

Beyond this direct interaction, the regulation of autophagy-related genes by estrogen relies heavily on transcription factors downstream of ESRs. As shown in Figure 3, upon estrogen stimulation, the expression of many autophagy-related genes is influenced by multiple transcription factors, each regulated by two or three ESRs. Complex interactions can occur among different receptors and transcription factors, as well as between transcription factors and autophagy-related genes. Notable examples include ESR1, ESR2, TFEB (transcription factor EB), ZKSCAN3 (zinc finger with KRAB and SCAN domain 3), and the tumor suppressor TP53 (tumor protein p53), along with MDM2 (MDM2 proto-oncogene) [105,112,113].

### 2.3. MicroRNAs in the Influence of Estrogen on Autophagy

In addition to the transcription factors previously discussed, estrogen (E2) regulates the expression of various autophagy-related proteins through microRNAs downstream of ESRs (Table S3) [111,114,115,116,117,118,119,120,121,122,123,124,125,126,127,128,129,130,131,132,133,134,135,136,137,138,139,140,141,142,143,144,145,146,147,148,149,150,151,152,153,154,155,156,157,158,159,160,161,162,163,164,165,166,167,168,169,170,171,172,173,174,175,176,177,178,179,180,181,182,183,184,185,186,187,188,189].

For instance, RB1CC1 (RB1 inducible coiled-coil 1), a nuclear DNA-binding protein that plays a crucial role in autophagy induction, is suppressed by *MIR10A-5p* (microRNA 10a) [171]. Notably, *MIR10A* expression can be upregulated by estrogen via ESR2 [173]. Similarly, *MIR17* suppresses the expression of ATG7 (autophagy-related 7) in human glioblastoma T98G cells [187]. In C2C12 myoblasts, *MIR20A* can inhibit ULK1 (unc-51 like autophagy activating kinase 1) expression [174]. Both *MIR17* and *MIR20A* are upregulated by E2 in MCF-7 cells through ESR1 signaling [175].

Moreover, *MIR21* has been identified as a regulator of BCL2 (BCL2 apoptosis regulator) mRNA in rat and human beta cells [180]. However, the impact of estrogen on *MIR21* expression varies with experimental conditions and cell lines; for example, E2 inhibits *MIR21* through ESR1 in HepG2 cells [181], while a selective agonist of ESR2 upregulates it in MCF-7 cells [178]. Additionally, both ATG12 and BECN1 are negatively regulated by STAT1 (signal transducer and activator of transcription 1) [184], which is also targeted by *MIR21* [131].

UCP2 (uncoupling protein 2), an inner mitochondrial membrane protein, can promote autophagy and endocrine resistance in breast cancer via the ROS1 (ROS proto-oncogene 1, receptor tyrosine kinase)–AKT1 (AKT serine/threonine kinase 1)–MTOR signaling pathway [48]. Estrogen treatment suppresses *Mir214* in ovariectomized mice and can directly inhibit UCP2 expression [176,190,191].

*HOTAIR* (HOX transcript antisense RNA) is a long non-coding RNA located within the homeobox C gene cluster. E2 induces *HOTAIR* expression by suppressing *MIR148A* through GPER1 [183]. *HOTAIR* has been implicated in regulating autophagy, influencing both cisplatin resistance and sensitivity to radiotherapy in various cancers [184,186,192]. In hepatocellular carcinoma, *HOTAIR* promotes autophagy by inducing the expression of ATG3 and ATG7 [186,188,193]. The transcription of *HOTAIR* upregulates ATG3 and ATG7, likely through an indirect competitive endogenous RNA mechanism, with sponging microRNAs contributing to the suppression of these genes [194].

Additionally, *HOTAIR* acts as a sponge for *MIR138-5p*, preventing its interaction with EZH2 (enhancer of zeste 2 polycomb repressive complex 2 subunit) and SIRT1 (sirtuin 1), thus enhancing the resistance of ovarian cancer cells to cisplatin-based chemotherapy [195]. In cholangiocarcinoma, *HOTAIR* suppresses autophagy by modulating the *MIR204-5p*–HMGB1 (high mobility group box 1) axis [182], while it regulates autophagy through the *MIR20B*-*5p*–ATG7 pathway in hepatic ischemia-reperfusion injury [196].

Furthermore, *HOTAIR* competes with *MIR17-5p* to regulate BECN1 expression [184,185,186,188,192,193,194,195,196] and to induce phosphorylation of SNAP23 (synaptosome-associated protein 23) via MTOR signaling [197]. In breast cancer, *HOTAIR* mediates autophagy progression through interactions matrix metallopeptidases, which are critical for cancer invasion; CTNNB1/β-catenin (catenin beta 1) may play a significant role in this process [198]. In a mouse model of Parkinson’s disease, *HOTAIR* drives autophagy in midbrain dopaminergic neurons by enhancing NPTX2 (neuronal pentraxin 2) expression through binding to *Mir221-3p* [189].

### 2.4. Histone Modifications in Estrogen’s Influence on Autophagy

**Figure 4:**
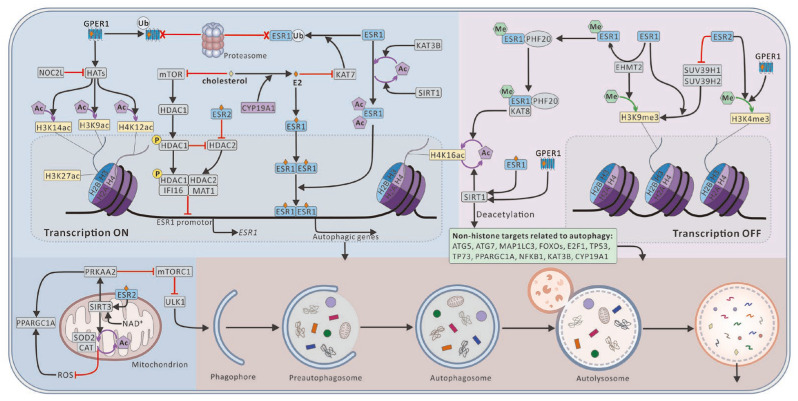
Estrogen regulation of core autophagy proteins via histone modifications. Sharp arrow (black), activates; blunt arrow (red), inhibits.

Histone acetylation and methylation play crucial roles in regulating gene transcription, acting as switches that affect overall chromatin structure. These modifications are likely involved in estrogen-dependent signaling pathways across various tissues in both normal and pathological states (Figure 4). Notably, cholesterol derivatives induce the dephosphorylation of HDAC1 (histone deacetylase 1), leading to the upregulation of autophagy [1]. Furthermore, suberoylanilide hydroxamic acid significantly inhibits the activities of HDAC1, HDAC2, and HDAC4, while downregulating GPER1 in a concentration-dependent manner [199].

SIRT1, an essential deacetylase, facilitates chromosomal condensation by mediating the loading of histone H1 and the condensin I complex onto chromosomes [200]. This process is critical in regulating the transcription of genes associated with various diseases. For instance, SIRT1 inhibits the puberty-activating gene *Kiss1* (KiSS-1 metastasis suppressor) by modulating the chromatin state at its promoter in KISS1/kisspeptin-neurokinin B-dynorphin neurons [201]. A deficiency in SIRT1 can lead to chromosomal instability and tumor progression. Additionally, estrogen upregulates SIRT1 expression through GPER1, subsequently activating the oncogenic EGFR (epidermal growth factor receptor) signaling pathway, which helps prevent apoptosis in human breast cancer cells, both in vitro and in vivo [202]. Importantly, SIRT1 deacetylates both histone and non-histone proteins and regulates estrogen biosynthesis by binding to multiple tissue-specific promoters, thereby enhancing the transcription of CYP19A1 (cytochrome P450 family 19 subfamily A member 1), which is also a target of SIRT1-mediated deacetylation [203,204].

Known histone targets of SIRT1 include H1 (Lys 26), H3 (Lys 9, Lys 19, and Lys 79), and H4 (Lys 8 and Lys 16) [200]. Notably, acetylation of histone H4 at Lys 16 serves as a primary substrate for SIRT1, influencing chromatin accessibility [113]. KAT8 (lysine acetyltransferase 8) primarily acetylates H4K16, along with certain non-histone proteins, and enhances the ESR1 signaling pathway through its acetylase activity in hepatocellular carcinoma [205].

Histone acetyltransferases (HATs) facilitate the conversion of the chromatin structure to a more relaxed and transcriptionally active state by acetylating specific lysine residues on histone tails [206]. HATs are pivotal in gene transcription and affect signaling cascades associated with inflammatory diseases, neurodegenerative disorders, and cancers through the acetylation of both histone and non-histone substrates [207,208]. Additionally, HATs have been implicated in promoting viral invasion and replication by interacting with viral proteins, such as the Tat protein of human immunodeficiency virus type 1 and the E1A protein of adenoviruses [209,210]. NOC2L (NOC2-like nucleolar-associated transcriptional repressor), an inhibitor of HATs, also regulates TP53 activity [207]. GPER1-mediated activation of HATs can lead to hyperacetylation of histones H3K9ac, H3K14ac, and H4K12ac [211]. In MCF-7 breast cancer cells, acetylated H3K27 promotes the transcription of ESR1 [212].

SIRT3 (sirtuin 3) functions as a mitochondrial nicotinamide adenine dinucleotide-dependent HDAC that promotes autophagy in lipotoxic hepatocytes via the AMP-activated protein kinase/ULK1 (unc-51 like autophagy activating kinase 1) pathway [128]. E2 can influence reactive oxygen species levels by activating SIRT3 via ESR2 in human seminoma TCam-2 cells. This mechanism is crucial for estrogen’s protective effects via ESR2 in adult germ cells, a function often lost in advanced tumors due to downregulation of ESR2 [129].

Moreover, in ovarian cancer, the tri-methylation of Lys4 on histone H3 is associated with ESR2, while levels of phosphorylated MAPK1 (mitogen-activated protein kinase 1)-MAPK3 are elevated through GPER1 activation [134,213].

The SIRT1–ESR1 signaling pathway plays a significant role in mitigating oxidative stress and neuroinflammation, as well as preventing arterial stiffness. This is achieved through enhanced signaling of the MAPK/JNK-NFKB1 (nuclear factor kappa B subunit 1) pathway and the upregulation of NOS3/eNOS (nitric oxide synthase 3) expression in various preclinical models of chronic diseases [214,215].

## 3. The Role of Autophagy and Feedback Loops in Estrogen Regulation

Estrogen, a hormone with significant physiological effects, is tightly regulated within the body. Cholesterol serves as the precursor for estrogen biosynthesis, and lipids released through autophagy provide a crucial source of cholesterol. Estrogen in the bloodstream can inhibit the release of hormones from glands through a negative feedback loop. Additionally, estrogen influences various histone modifications, transcription factors, and microRNAs that regulate both estrogen biosynthesis and ESRs, creating further negative feedback loops to finely tune estrogen-mediated cellular responses, including autophagy [178,216].

Regarding the fate of ESRs, human cells utilize both the ubiquitin–proteasome system and autophagy pathways for proteolysis. In eukaryotic cells, ESR1 is primarily degraded through the ubiquitin–proteasome system; however, its levels also decrease with autophagosome formation [48]. This aligns with prior findings on ESR1 degradation via autophagy, highlighting the inhibitory effects of E2 and other GPER1 agonists on cellular autophagy [18]. Meanwhile, ESR2 localizes to mitochondria, which are significant substrates for autophagy. GPER1 has also been observed at the autophagosome membrane, showing considerable colocalization with MAP1LC3 [11]. However, it appears that GPER1 may not be a substrate for autophagy, as its expression levels do not decrease with increased autophagy activity [13], though further research is necessary to confirm this.

## 4. Concluding Remarks and Perspectives

Both autophagy promoters and inhibitors have shown anticancer effects in vitro, yet individual responses in clinical trials vary significantly [217,218]. Estrogen plays a crucial role in these differences, influencing cancer treatment outcomes [219,220]. Its relationship with cancer is complex; for instance, while estrogen can delay apoptosis in ESR1-positive breast cancer cells, it may also protect some women against the disease at a circulatory level and induce apoptosis in estrogen-resistant cancers [12,221,222]. Similar to anticancer trials on autophagy modulators, several studies have examined the effectiveness of combining estrogen or its antagonist, raloxifene, with autophagy inducers (including rapamycin and everolimus) or inhibitors (such as chloroquine and hydroxychloroquine) alongside anti-infection therapies, including those targeting SARS-CoV-2 [223,224,225,226,227,228].

Our discussion highlights that estrogen promotes autophagy through estrogen receptors by regulating autophagy-related proteins via downstream transcription factors, miRNAs, and histone modifications. Consequently, the effects of estrogen on autophagy can vary with estrogen levels and the expression of its receptors. Autophagy also influences estrogen, with cholesterol produced through autophagy serving as a precursor for estrogen synthesis, while excessive autophagy may degrade estrogen receptors.

**Figure 5:**
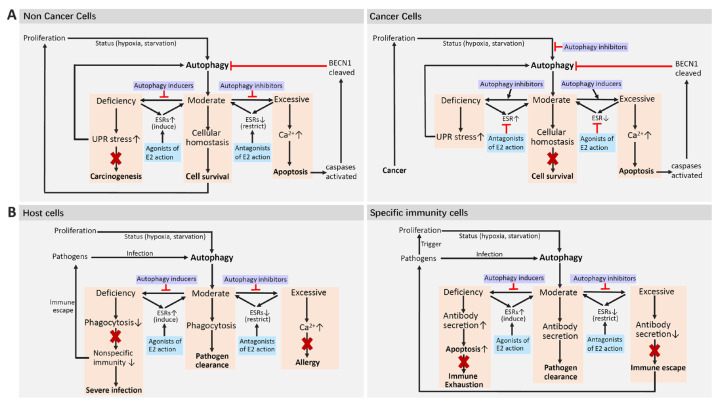
Model of the effects of the estrogen–autophagy axis on cell fate in cancers and infectious diseases. (**A**) For the prevention and treatment of cancer, cells can be divided into non-cancer and cancer cells. A moderate status of autophagy represents cellular homeostasis, which is beneficial for cancer prevention, but detrimental for cancer treatment. Deficient autophagy results in stress via the unfolded protein response (UPR), which may induce autophagy to re-establish homeostasis or further result in carcinogenesis. Excessive autophagy releases excess calcium, which triggers apoptosis via activated caspases. Both E2 agonists and autophagy inducers can prevent carcinogenesis when autophagy is deficient in non-cancer cells or apoptosis of cancer cells via excessive autophagy. Both E2 antagonists and autophagy inhibitors seem to inhibit apoptosis of non-cancer cells and disrupt homeostasis of cancer cells. (**B**) For the prevention and treatment of pathogen infections, cells can be divided into host cells and specialized immune cells. In host cells, a moderate status of autophagy represents moderate phagocytosis for pathogen clearance. Deficient autophagy may lead to immune escape and further result in severe infection. Excessive autophagy releases excess calcium, which can trigger allergies. In specialized immune cells, a moderate status of autophagy corresponds to moderate release of antibodies to act on infected target cells and pathogen clearance. Insufficient autophagy will accelerate the release of antibodies and cause rapid apoptosis of immune cells, while excessive autophagy will reduce the release of antibodies and cause immune escape. Sharp arrow (black), activates; blunt arrow (red), inhibits.

To clarify the interplay between estrogen and autophagy in cancer progression, we propose a new model (Figure 5), aligned with previous models emphasizing autophagy’s role in maintaining intracellular quality control and homeostasis. This model suggests that estrogen primarily enhances autophagy by regulating ESR levels to ensure balanced autophagy and cellular stability, contrasting with earlier views of estrogen’s dual protective effects.

In our model, autophagy is closely linked to ESR levels: excessive autophagy correlates with lower ESR levels, while insufficient autophagy is associated with higher levels. Effective anticancer therapies may disrupt the homeostasis of cancer cells while preserving normal cells. For ESR1-positive breast cancers, estrogen antagonism might be necessary due to the connection between insufficient autophagy and elevated ESR levels, while ESR1-negative cancers might respond better to estrogen agonists.

Estrogen can upregulate ESR1, ESR2, and GPER1 [229,230], but its levels are tightly regulated by negative feedback loops and can fluctuate due to factors such as age, sex, menstrual cycle, and environmental influences [231,232,233,234,235]. This variability indicates that autophagy is also susceptible to these factors. Our model suggests that ESR levels may serve as indirect indicators of a cell’s autophagic state, although further research is needed.

Both autophagy modulators (rapamycin and everolimus) and inhibitors (chloroquine and hydroxychloroquine) have demonstrated anti-infection properties against various pathogens, including SARS-CoV-2 [225,226,227,228]. Just as there are individual differences in cancer trials, variations also exist in trials involving microbial pathogens. Recent findings suggest that manipulating estrogen’s effects on autophagy could lead to new treatment strategies for various diseases, not just cancer. Notably, estrogen may interfere with treatments for infections and cancer immunotherapy by regulating the autophagy of immune cells, including macrophages as well as T and B lymphocytes [63,70,142,151,157], which also explains, to some extent, individual differences in responses to autophagy modulators in the anti-infection treatment (Figure 5B).

Overall, our findings indicate a much closer relationship between estrogen and autophagy than previously recognized. This association affects numerous biological processes, aiding in the understanding of disease mechanisms and the development of innovative treatment approaches, and underscores the need for continued research in this area.

## Data Availability

Data are contained within the article and Supplementary Materials.

## Supplementary Materials

The following supporting information can be downloaded at: https://www.mdpi.com/article/10.3390/ijms252312576/s1.

## References

[1] Wu W., Luo M., Li K., Dai Y., Yi H., Zhong Y., Cao Y. (2021). Cholesterol derivatives induce dephosphorylation of the histone deacetylases Rpd3/HDAC1 to upregulate autophagy. Autophagy.

[2] Zhao Z.Q., Song W., Yan X.Q., Tang J.H., Hou J.C., Wang D.D., Yang S.J., Zhang Q., Zhang J. (2021). Autophagy Modulation and Synergistic Therapy to Combat Multidrug Resistance Breast Cancer Using Hybrid Cell Membrane Nanoparticles. J. Biomed. Nanotechnol..

[3] Khan N., Chen X., Geiger J.D. (2021). Possible Therapeutic Use of Natural Compounds Against COVID-19. J. Cell. Signal.

[4] Ulgherait M., Midoun A.M., Park S.J., Gatto J.A., Tener S.J., Siewert J., Klickstein N., Canman J.C., Ja W.W., Shirasu-Hiza M. (2021). Circadian autophagy drives iTRF-mediated longevity. Nature.

[5] Di Vincenzo A., Andrisani A., Vettor R., Rossato M. (2021). Estrogen and COVID-19: Friend or foe?. Ann. Oncol..

[6] Jia M., Dahlman-Wright K., Gustafsson J.Å. (2015). Estrogen receptor alpha and beta in health and disease. Best. Pract. Res. Clin. Endocrinol. Metab..

[7] Zhang B., Liu L. (2021). Autophagy is a double-edged sword in the therapy of colorectal cancer. Oncol. Lett..

[8] Zhang Z., Shi J., Nice E.C., Huang C., Shi Z. (2021). The Multifaceted Role of Flavonoids in Cancer Therapy: Leveraging Autophagy with a Double-Edged Sword. Antioxidants.

[9] Xiang J., Liu X., Ren J., Chen K., Wang H.L., Miao Y.Y., Qi M.M. (2019). How does estrogen work on autophagy?. Autophagy.

[10] Abderrahman B., Maximov P.Y., Curpan R.F., Hanspal J.S., Fan P., Xiong R., Tonetti D.A., Thatcher G.R.J., Jordan V.C. (2020). Pharmacology and Molecular Mechanisms of Clinically Relevant Estrogen Estetrol and Estrogen Mimic BMI-135 for the Treatment of Endocrine-Resistant Breast Cancer. Mol. Pharmacol..

[11] Qi M., Liu X., Zhou Y., Wang H., Zhao Y., Ren J., Xiang J. (2021). Berberine Inhibits MDA-MB-231 Cells as an Agonist of G Protein-Coupled Estrogen Receptor 1. Int. J. Mol. Sci..

[12] Lønning P.E., Taylor P.D., Anker G., Iddon J., Wie L., Jørgensen L.M., Mella O., Howell A. (2001). High-dose estrogen treatment in postmenopausal breast cancer patients heavily exposed to endocrine therapy. Breast Cancer Res. Treat..

[13] Pei H., Wang W., Zhao D., Su H., Su G., Zhao Z. (2019). G Protein-Coupled Estrogen Receptor 1 Inhibits Angiotensin II-Induced Cardiomyocyte Hypertrophy via the Regulation of PI3K-Akt-mTOR Signalling and Autophagy. Int. J. Biol. Sci..

[14] Song S., Wu S., Wang Y., Wang Z., Ye C., Song R., Song D., Ruan Y. (2018). 17β-estradiol inhibits human umbilical vascular endothelial cell senescence by regulating autophagy via p53. Exp. Gerontol..

[15] Park J., Shin H., Song H., Lim H.J. (2016). Autophagic regulation in steroid hormone-responsive systems. Steroids.

[16] Schiebler T.H., Danner K.G. (1978). The effect of sex hormones on the proximal tubules in the rat kidney. Cell Tissue Res..

[17] Meyer M.R., Barton M. (2016). Estrogens and Coronary Artery Disease: New Clinical Perspectives. Adv. Pharmacol..

[18] Totta P., Busonero C., Leone S., Marino M., Acconcia F. (2016). Dynamin II is required for 17β-estradiol signaling and autophagy-based ERα degradation. Sci. Rep..

[19] Song P., Li Y., Dong Y., Liang Y., Qu H., Qi D., Lu Y., Jin X., Guo Y., Jia Y. (2019). Estrogen receptor β inhibits breast cancer cells migration and invasion through CLDN6-mediated autophagy. J. Exp. Clin. Cancer Res..

[20] Lahm T., Petrache I. (2012). LC3 as a potential therapeutic target in hypoxia-induced pulmonary hypertension. Autophagy.

[21] Lahm T., Albrecht M., Fisher A.J., Selej M., Patel N.G., Brown J.A., Justice M.J., Brown M.B., Van Demark M., Trulock K.M. (2012). 17β-Estradiol attenuates hypoxic pulmonary hypertension via estrogen receptor-mediated effects. Am. J. Respir. Crit. Care Med..

[22] Ao P., Huang W., Li J., Wu T., Xu L., Deng Z., Chen W., Yin C., Cheng X. (2018). 17β-estradiol protects nucleus pulposus cells from serum deprivation-induced apoptosis and regulates expression of MMP-3 and MMP-13 through promotion of autophagy. Biochem. Biophys. Res. Commun..

[23] Florencio-Silva R., Sasso G.R.S., Sasso-Cerri E., Simões M.J., Cerri P.S. (2018). Effects of estrogen status in osteocyte autophagy and its relation to osteocyte viability in alveolar process of ovariectomized rats. Biomed. Pharmacother..

[24] Yang Y.H., Chen K., Li B., Chen J.W., Zheng X.F., Wang Y.R., Jiang S.D., Jiang L.S. (2013). Estradiol inhibits osteoblast apoptosis via promotion of autophagy through the ER-ERK-mTOR pathway. Apoptosis.

[25] Sun X., Yang X., Zhao Y., Li Y., Guo L. (2018). Effects of 17β-Estradiol on Mitophagy in the Murine MC3T3-E1 Osteoblast Cell Line is Mediated via G Protein-Coupled Estrogen Receptor and the ERK1/2 Signaling Pathway. Med. Sci. Monit..

[26] Gavali S., Gupta M.K., Daswani B., Wani M.R., Sirdeshmukh R., Khatkhatay M.I. (2019). Estrogen enhances human osteoblast survival and function via promotion of autophagy. Biochim. Biophys. Acta Mol. Cell Res..

[27] Li X.Z., Sui C.Y., Chen Q., Zhuang Y.S., Zhang H., Zhou X.P. (2016). The effects and mechanism of estrogen on rats with Parkinson’s disease in different age groups. Am. J. Transl. Res..

[28] Chen K.C., Lin C.M., Huang C.J., Chen S.K., Wu S.T., Chiang H.S., Ku W.C. (2016). Dual Roles of 17-β Estradiol in Estrogen Receptor-dependent Growth Inhibition in Renal Cell Carcinoma. Cancer Genom. Proteom..

[29] Wei Q., Liang X., Peng Y., Yu D., Zhang R., Jin H., Fan J., Cai W., Ren C., Yu J. (2018). 17β-estradiol ameliorates oxidative stress and blue light-emitting diode-induced retinal degeneration by decreasing apoptosis and enhancing autophagy. Drug Des. Dev. Ther..

[30] Guido C., Panza S., Santoro M., Avena P., Panno M.L., Perrotta I., Giordano F., Casaburi I., Catalano S., De Amicis F. (2012). Estrogen receptor beta (ERβ) produces autophagy and necroptosis in human seminoma cell line through the binding of the Sp1 on the phosphatase and tensin homolog deleted from chromosome 10 (PTEN) promoter gene. Cell Cycle.

[31] Mei J., Zhou W.J., Zhu X.Y., Lu H., Wu K., Yang H.L., Fu Q., Wei C.Y., Chang K.K., Jin L.P. (2018). Suppression of autophagy and HCK signaling promotes PTGS2^high^ FCGR3^−^ NK cell differentiation triggered by ectopic endometrial stromal cells. Autophagy.

[32] Hsieh D.J., Kuo W.W., Lai Y.P., Shibu M.A., Shen C.Y., Pai P., Yeh Y.L., Lin J.Y., Viswanadha V.P., Huang C.Y. (2015). 17β-Estradiol and/or Estrogen Receptor β Attenuate the Autophagic and Apoptotic Effects Induced by Prolonged Hypoxia Through HIF-1α-Mediated BNIP3 and IGFBP-3 Signaling Blockage. Cell. Physiol. Biochem..

[33] Wei Q., Zhu R., Zhu J., Zhao R., Li M. (2019). E2-Induced Activation of the NLRP3 Inflammasome Triggers Pyroptosis and Inhibits Autophagy in HCC Cells. Oncol. Res..

[34] Jin L.Y., Lv Z.D., Wang K., Qian L., Song X.X., Li X.F., Shen H.X. (2018). Estradiol Alleviates Intervertebral Disc Degeneration through Modulating the Antioxidant Enzymes and Inhibiting Autophagy in the Model of Menopause Rats. Oxid. Med. Cell. Longev..

[35] Li L., Chen J., Sun S., Zhao J., Dong X., Wang J. (2017). Effects of Estradiol on Autophagy and Nrf-2/ARE Signals after Cerebral Ischemia. Cell. Physiol. Biochem..

[36] Wang F., Xiao J., Shen Y., Yao F., Chen Y. (2014). Estrogen protects cardiomyocytes against lipopolysaccharide by inhibiting autophagy. Mol. Med. Rep..

[37] Kimura A., Ishida Y., Nosaka M., Kuninaka Y., Hama M., Kawaguchi T., Sakamoto S., Shinozaki K., Iwahashi Y., Takayasu T. (2016). Exaggerated arsenic nephrotoxicity in female mice through estrogen-dependent impairments in the autophagic flux. Toxicology.

[38] Yang Y., Zheng X., Li B., Jiang S., Jiang L. (2014). Increased activity of osteocyte autophagy in ovariectomized rats and its correlation with oxidative stress status and bone loss. Biochem. Biophys. Res. Commun..

[39] Lin C.W., Chen B., Huang K.L., Dai Y.S., Teng H.L. (2016). Inhibition of Autophagy by Estradiol Promotes Locomotor Recovery after Spinal Cord Injury in Rats. Neurosci. Bull..

[40] Tao Z., Zheng L.D., Smith C., Luo J., Robinson A., Almeida F.A., Wang Z., Olumi A.F., Liu D., Cheng Z. (2018). Estradiol signaling mediates gender difference in visceral adiposity via autophagy. Cell Death Dis..

[41] Klionsky D.J., Abdel-Aziz A.K., Abdelfatah S., Abdellatif M., Abdoli A., Abel S., Abeliovich H., Abildgaard M.H., Abudu Y.P., Acevedo-Arozena A. (2021). Guidelines for the use and interpretation of assays for monitoring autophagy (4th edition)^1^. Autophagy.

[42] Bai L.Y., Weng J.R., Hu J.L., Wang D., Sargeant A.M., Chiu C.F. (2013). G15, a GPR30 antagonist, induces apoptosis and autophagy in human oral squamous carcinoma cells. Chem. Biol. Interact..

[43] Peng Y.Q., Xiong D., Lin X., Cui R.R., Xu F., Zhong J.Y., Zhu T., Wu F., Mao M.Z., Liao X.B. (2017). Oestrogen Inhibits Arterial Calcification by Promoting Autophagy. Sci. Rep..

[44] Yue J., Wang X.S., Feng B., Hu L.N., Yang L.K., Lu L., Zhang K., Wang Y.T., Liu S.B. (2019). Activation of G-Protein-Coupled Receptor 30 Protects Neurons against Excitotoxicity through Inhibiting Excessive Autophagy Induced by Glutamate. ACS Chem. Neurosci..

[45] Pawlik A., Słomińska-Wojewódzka M., Herman-Antosiewicz A., Antosiewicz A. (2016). Sensitization of estrogen receptor-positive breast cancer cell lines to 4-hydroxytamoxifen by isothiocyanates present in cruciferous plants. Eur. J. Nutr..

[46] Duan L., Danzer B., Levenson V.V., Maki C.G. (2014). Critical roles for nitric oxide and ERK in the completion of prosurvival autophagy in 4OHTAM-treated estrogen receptor-positive breast cancer cells. Cancer Lett..

[47] Yu X., Li R., Shi W., Jiang T., Wang Y., Li C., Qu X. (2016). Silencing of MicroRNA-21 confers the sensitivity to tamoxifen and fulvestrant by enhancing autophagic cell death through inhibition of the PI3K-AKT-mTOR pathway in breast cancer cells. Biomed. Pharmacother..

[48] Yu X., Luo A., Liu Y., Wang S., Li Y., Shi W., Liu Z., Qu X. (2015). MiR-214 increases the sensitivity of breast cancer cells to tamoxifen and fulvestrant through inhibition of autophagy. Mol. Cancer.

[49] Li L., Li X., Han X., Yang T., Fu J., Zhang Y., Gou W. (2014). An ovarian cancer model with positive ER: Reversion of ER antagonist resistance by Src blockade. Oncol. Rep..

[50] Simpkins F., Hevia-Paez P., Sun J., Ullmer W., Gilbert C.A., da Silva T., Pedram A., Levin E.R., Reis I.M., Rabinovich B. (2012). Src Inhibition with saracatinib reverses fulvestrant resistance in ER-positive ovarian cancer models in vitro and in vivo. Clin. Cancer Res..

[51] Zhou W.J., Zhang J., Yang H.L., Wu K., Xie F., Wu J.N., Wang Y., Yao L., Zhuang Y., Xiang J.D. (2019). Estrogen inhibits autophagy and promotes growth of endometrial cancer by promoting glutamine metabolism. Cell Commun. Signal.

[52] Alayev A., Berger S.M., Kramer M.Y., Schwartz N.S., Holz M.K. (2015). The combination of rapamycin and resveratrol blocks autophagy and induces apoptosis in breast cancer cells. J. Cell. Biochem..

[53] Turan B., Tuncay E., Vassort G. (2012). Resveratrol and diabetic cardiac function: Focus on recent in vitro and in vivo studies. J. Bioenerg. Biomembr..

[54] Zhang X., Xu W., Su J., Chu M., Jin H., Li G., Tan C., Wang X., Wang C. (2014). The prosurvival role of autophagy in resveratrol-induced cytotoxicity in GH3 cells. Int. J. Mol. Med..

[55] Zhou F., Dong H., Liu Y., Yan L., Sun C., Hao P., Liu Y., Zhai J., Liu Y. (2018). Raloxifene, a promising estrogen replacement, limits TDP-25 cell death by enhancing autophagy and suppressing apoptosis. Brain Res. Bull..

[56] Kim D.E., Kim Y., Cho D.H., Jeong S.Y., Kim S.B., Suh N., Lee J.S., Choi E.K., Koh J.Y., Hwang J.J. (2015). Raloxifene induces autophagy-dependent cell death in breast cancer cells via the activation of AMP-activated protein kinase. Mol. Cells.

[57] Wu S.T., Sun G.H., Cha T.L., Kao C.C., Chang S.Y., Kuo S.C., Way T.D. (2016). CSC-3436 switched tamoxifen-induced autophagy to apoptosis through the inhibition of AMPK/mTOR pathway. J. Biomed. Sci..

[58] Liu Z.R., Song Y., Wan L.H., Zhang Y.Y., Zhou L.M. (2016). Over-expression of miR-451a can enhance the sensitivity of breast cancer cells to tamoxifen by regulating 14-3-3ζ, estrogen receptor α, and autophagy. Life Sci..

[59] Kim H.S., Tian L., Jung M., Choi S.K., Sun Y., Kim H., Moon W.K. (2015). Downregulation of Choline Kinase-Alpha Enhances Autophagy in Tamoxifen-Resistant Breast Cancer Cells. PLoS ONE.

[60] Nagelkerke A., Sieuwerts A.M., Bussink J., Sweep F.C., Look M.P., Foekens J.A., Martens J.W., Span P.N. (2014). LAMP3 is involved in tamoxifen resistance in breast cancer cells through the modulation of autophagy. Endocr. Relat. Cancer.

[61] Graham C.D., Kaza N., Klocke B.J., Gillespie G.Y., Shevde L.A., Carroll S.L., Roth K.A. (2016). Tamoxifen Induces Cytotoxic Autophagy in Glioblastoma. J. Neuropathol. Exp. Neurol..

[62] Dittmar A.J., Drozda A.A., Blader I.J. (2016). Drug Repurposing Screening Identifies Novel Compounds That Effectively Inhibit Toxoplasma gondii Growth. mSphere.

[63] Pierdominici M., Maselli A., Locatelli S.L., Ciarlo L., Careddu G., Patrizio M., Ascione B., Tinari A., Carlo-Stella C., Malorni W. (2017). Estrogen receptor β ligation inhibits Hodgkin lymphoma growth by inducing autophagy. Oncotarget.

[64] Yang Z., Yu W., Liu B., Yang M., Tao H. (2020). Estrogen receptor β induces autophagy of osteosarcoma through the mTOR signaling pathway. J. Orthop. Surg. Res..

[65] Ruddy S.C., Lau R., Cabrita M.A., McGregor C., McKay B.C., Murphy L.C., Wright J.S., Durst T., Pratt M.A. (2014). Preferential estrogen receptor β ligands reduce Bcl-2 expression in hormone-resistant breast cancer cells to increase autophagy. Mol. Cancer Ther..

[66] Bravo D., Shogren K.L., Zuo D., Wagner E.R., Sarkar G., Yaszemski M.J., Maran A. (2017). 2-Methoxyestradiol-Mediated Induction of Frzb Contributes to Cell Death and Autophagy in MG63 Osteosarcoma Cells. J. Cell. Biochem..

[67] Liu C., Zhou X., Lu J., Zhu L., Li M. (2019). Autophagy mediates 2-methoxyestradiol-inhibited scleroderma collagen synthesis and endothelial-to-mesenchymal transition induced by hypoxia. Rheumatology.

[68] Lee M.J., Kim E.H., Lee S.A., Kang Y.M., Jung C.H., Yoon H.K., Seol S.M., Lee Y.L., Lee W.J., Park J.Y. (2015). Dehydroepiandrosterone prevents linoleic acid-induced endothelial cell senescence by increasing autophagy. Metabolism.

[69] Rovito D., Giordano C., Vizza D., Plastina P., Barone I., Casaburi I., Lanzino M., De Amicis F., Sisci D., Mauro L. (2013). Omega-3 PUFA ethanolamides DHEA and EPEA induce autophagy through PPARγ activation in MCF-7 breast cancer cells. J. Cell. Physiol..

[70] Bongiovanni B., Mata-Espinosa D., D’Attilio L., Leon-Contreras J.C., Marquez-Velasco R., Bottasso O., Hernandez-Pando R., Bay M.L. (2015). Effect of cortisol and/or DHEA on THP1-derived macrophages infected with Mycobacterium tuberculosis. Tuberculosis.

[71] Prietsch R.F., Monte L.G., da Silva F.A., Beira F.T., Del Pino F.A., Campos V.F., Collares T., Pinto L.S., Spanevello R.M., Gamaro G.D. (2014). Genistein induces apoptosis and autophagy in human breast MCF-7 cells by modulating the expression of proapoptotic factors and oxidative stress enzymes. Mol. Cell. Biochem..

[72] Backes F.J., Walker C.J., Goodfellow P.J., Hade E.M., Agarwal G., Mutch D., Cohn D.E., Suarez A.A. (2016). Estrogen receptor-alpha as a predictive biomarker in endometrioid endometrial cancer. Gynecol. Oncol..

[73] Jorns J.M. (2019). Breast Cancer Biomarkers: Challenges in Routine Estrogen Receptor, Progesterone Receptor, and HER2/neu Evaluation. Arch. Pathol. Lab. Med..

[74] Matsuo K., Sheridan T.B., Mabuchi S., Yoshino K., Hasegawa K., Studeman K.D., Im D.D., Rosenshein N.B., Roman L.D., Sood A.K. (2014). Estrogen receptor expression and increased risk of lymphovascular space invasion in high-grade serous ovarian carcinoma. Gynecol. Oncol..

[75] Haydar Ali Tajuddin A., Kamaruddin N., Sukor N., Azizan E.A., Omar A.M. (2020). Estrogen Receptors in Nonfunctioning Pituitary Neuroendocrine Tumors: Review on Expression and Gonadotroph Functions. J. Endocr. Soc..

[76] Liu J.Y.H., Lin G., Fang M., Rudd J.A. (2019). Localization of estrogen receptor ERα, ERβ and GPR30 on myenteric neurons of the gastrointestinal tract and their role in motility. Gen. Comp. Endocrinol..

[77] Dijksterhuis W.P.M., Kalff M.C., Wagner A.D., Verhoeven R.H.A., Lemmens V.E.P.P., van Oijen M.G.H., Gisbertz S.S., van Berge Henegouwen M.I., van Laarhoven H.W.M. (2021). Gender Differences in Treatment Allocation and Survival of Advanced Gastroesophageal Cancer: A Population-Based Study. J. Natl. Cancer Inst..

[78] Lee J.Y., Kim N., Park J.H., Yu J.E., Song Y.J., Yoon J.W., Lee D.H. (2022). Sex and Gender Differences in Overlap Syndrome of Functional Gastrointestinal Disorder and Effect of Genetic Polymorphisms in South Korea: A Long-term Follow-up Study. J. Neurogastroenterol. Motil..

[79] Chen L.R., Ko N.Y., Chen K.H. (2019). Isoflavone Supplements for Menopausal Women: A Systematic Review. Nutrients.

[80] Goto T., Miyamoto H. (2021). The Role of Estrogen Receptors in Urothelial Cancer. Front. Endocrinol..

[81] Sen A., Kaul A., Kaul R. (2021). Estrogen receptors in human bladder cells regulate innate cytokine responses to differentially modulate uropathogenic E. coli colonization. Immunobiology.

[82] Uebi T., Umeda M., Imai T. (2015). Estrogen induces estrogen receptor alpha expression and hepatocyte proliferation in the livers of male mice. Genes. Cells.

[83] Iñigo M.R., Amorese A.J., Tarpey M.D., Balestrieri N.P., Jones K.G., Patteson D.J., Jackson K.C., Torres M.J., Lin C.T., Smith C.D. (2020). Estrogen receptor-α in female skeletal muscle is not required for regulation of muscle insulin sensitivity and mitochondrial regulation. Mol. Metab..

[84] Seko D., Fujita R., Kitajima Y., Nakamura K., Imai Y., Ono Y. (2020). Estrogen Receptor β Controls Muscle Growth and Regeneration in Young Female Mice. Stem Cell Rep..

[85] Zhang B., Zhang C.G., Ji L.H., Zhao G., Wu Z.Y. (2018). Estrogen receptor β selective agonist ameliorates liver cirrhosis in rats by inhibiting the activation and proliferation of hepatic stellate cells. J. Gastroenterol. Hepatol..

[86] Chen C., Gong X., Yang X., Shang X., Du Q., Liao Q., Xie R., Chen Y., Xu J. (2019). The roles of estrogen and estrogen receptors in gastrointestinal disease. Oncol. Lett..

[87] Kawabata W., Suzuki T., Moriya T., Fujimori K., Naganuma H., Inoue S., Kinouchi Y., Kameyama K., Takami H., Shimosegawa T. (2003). Estrogen receptors (alpha and beta) and 17beta-hydroxysteroid dehydrogenase type 1 and 2 in thyroid disorders: Possible in situ estrogen synthesis and actions. Mod. Pathol..

[88] Morani A., Barros R.P., Imamov O., Hultenby K., Arner A., Warner M., Gustafsson J.A. (2006). Lung dysfunction causes systemic hypoxia in estrogen receptor beta knockout (ERbeta-/-) mice. Proc. Natl. Acad. Sci. USA.

[89] Hsu L.H., Chu N.M., Kao S.H. (2017). Estrogen, Estrogen Receptor and Lung Cancer. Int. J. Mol. Sci..

[90] Välimaa H., Savolainen S., Soukka T., Silvoniemi P., Mäkelä S., Kujari H., Gustafsson J.A., Laine M. (2004). Estrogen receptor-beta is the predominant estrogen receptor subtype in human oral epithelium and salivary glands. J. Endocrinol..

[91] Grimm M., Biegner T., Teriete P., Hoefert S., Krimmel M., Munz A., Reinert S. (2016). Estrogen and Progesterone hormone receptor expression in oral cavity cancer. Med. Oral Patol. Oral Cir. Bucal.

[92] Tropea T., De Francesco E.M., Rigiracciolo D., Maggiolini M., Wareing M., Osol G., Mandalà M. (2015). Pregnancy Augments G Protein Estrogen Receptor (GPER) Induced Vasodilation in Rat Uterine Arteries via the Nitric Oxide—cGMP Signaling Pathway. PLoS ONE.

[93] Sharma G., Prossnitz E.R. (2017). G-Protein-Coupled Estrogen Receptor (GPER) and Sex-Specific Metabolic Homeostasis. Adv. Exp. Med. Biol..

[94] Camilletti M.A., Abeledo-Machado A., Ferraris J., Pérez P.A., Faraoni E.Y., Pisera D., Gutierrez S., Díaz-Torga G. (2019). Role of GPER in the anterior pituitary gland focusing on lactotroph function. J. Endocrinol..

[95] Li R., Wang Y., Chen P., Meng J., Zhang H. (2021). Inhibiting endoplasmic reticulum stress by activation of G-protein-coupled estrogen receptor to protect retinal astrocytes under hyperoxia. J. Biochem. Mol. Toxicol..

[96] Della Torre S. (2020). Non-alcoholic Fatty Liver Disease as a Canonical Example of Metabolic Inflammatory-Based Liver Disease Showing a Sex-Specific Prevalence: Relevance of Estrogen Signaling. Front. Endocrinol..

[97] Fredette N.C., Meyer M.R., Prossnitz E.R. (2018). Role of GPER in estrogen-dependent nitric oxide formation and vasodilation. J. Steroid Biochem. Mol. Biol..

[98] Triplett K.D., Pokhrel S., Castleman M.J., Daly S.M., Elmore B.O., Joyner J.A., Sharma G., Herbert G., Campen M.J., Hathaway H.J. (2019). GPER activation protects against epithelial barrier disruption by Staphylococcus aureus α-toxin. Sci. Rep..

[99] Zhou L., Yu T., Yang F., Han J., Zuo B., Huang L., Bai X., Jiang M., Wu D., Chen S. (2021). G Protein-Coupled Estrogen Receptor Agonist G-1 Inhibits Mantle Cell Lymphoma Growth in Preclinical Models. Front. Oncol..

[100] Bertoni A.P.S., Manfroi P.A., Tomedi J., Assis-Brasil B.M., de Souza Meyer E.L., Furlanetto T.W. (2021). The gene expression of GPER1 is low in fresh samples of papillary thyroid carcinoma (PTC), and in silico analysis. Mol. Cell. Endocrinol..

[101] Tang C., Yang L., Wang N., Li L., Xu M., Chen G.G., Liu Z.M. (2014). High expression of GPER1, EGFR and CXCR1 is associated with lymph node metastasis in papillary thyroid carcinoma. Int. J. Clin. Exp. Pathol..

[102] Shen Y., Li C., Zhou L., Huang J.A., Shen Y., Li C., Zhou L., Huang J.A. (2021). G protein-coupled oestrogen receptor promotes cell growth of non-small cell lung cancer cells via YAP1/QKI/circNOTCH1/m6A methylated NOTCH1 signalling. J. Cell. Mol. Med..

[103] Chen Z.J., Wei W., Jiang G.M., Liu H., Wei W.D., Yang X., Wu Y.M., Liu H., Wong C.K., Du J. (2016). Activation of GPER suppresses epithelial mesenchymal transition of triple negative breast cancer cells via NF-κB signals. Mol. Oncol..

[104] Tian S., Zhan N., Li R., Dong W. (2019). Downregulation of G Protein-Coupled Estrogen Receptor (GPER) is Associated with Reduced Prognosis in Patients with Gastric Cancer. Med. Sci. Monit..

[105] Muller C., Brown-Glaberman U.A., Chaney M.F., Garyantes T., LoRusso P., McQuade J.L., Mita A.C., Mita M.M., Natale C., Orloff M. (2021). Phase 1 trial of a novel, first-in-class G protein-coupled estrogen receptor (GPER) agonist, LNS8801, in patients with advanced or recurrent treatment-refractory solid malignancies. J. Clin. Oncol..

[106] Filardo E., Quinn J., Pang Y., Graeber C., Shaw S., Dong J., Thomas P. (2007). Activation of the novel estrogen receptor G protein-coupled receptor 30 (GPR30) at the plasma membrane. Endocrinology.

[107] Otto C., Rohde-Schulz B., Schwarz G., Fuchs I., Klewer M., Brittain D., Langer G., Bader B., Prelle K., Nubbemeyer R. (2008). G protein-coupled receptor 30 localizes to the endoplasmic reticulum and is not activated by estradiol. Endocrinology.

[108] Razandi M., Alton G., Pedram A., Ghonshani S., Webb P., Levin E.R. (2003). Identification of a structural determinant necessary for the localization and function of estrogen receptor alpha at the plasma membrane. Mol. Cell. Biol..

[109] Thomas P., Pang Y., Filardo E.J., Dong J. (2005). Identity of an estrogen membrane receptor coupled to a G protein in human breast cancer cells. Endocrinology.

[110] Alayev A., Salamon R.S., Berger S.M., Schwartz N.S., Cuesta R., Snyder R.B., Holz M.K. (2016). mTORC1 directly phosphorylates and activates ERα upon estrogen stimulation. Oncogene.

[111] Jerjees D.A., Negm O.H., Alabdullah M.L., Mirza S., Alkaabi M., Hameed M.R., Abduljabbar R., Muftah A., Nolan C.C., Green A.R. (2015). The mammalian target of rapamycin complex 1 (mTORC1) in breast cancer: The impact of oestrogen receptor and HER2 pathways. Breast Cancer Res. Treat..

[112] Caldon C.E. (2014). Estrogen signaling and the DNA damage response in hormone dependent breast cancers. Front. Oncol..

[113] Füllgrabe J., Klionsky D.J., Joseph B. (2014). The return of the nucleus: Transcriptional and epigenetic control of autophagy. Nat. Rev. Mol. Cell Biol..

[114] Wu Y.F., Li Z.Y., Dong L.L., Li W.J., Wu Y.P., Wang J., Chen H.P., Liu H.W., Li M., Jin C.L. (2020). Inactivation of MTOR promotes autophagy-mediated epithelial injury in particulate matter-induced airway inflammation. Autophagy.

[115] Kalaitzidis D., Gilmore T.D. (2005). Transcription factor cross-talk: The estrogen receptor and NF-kappaB. Trends Endocrinol. Metab..

[116] Liang S., Chen Z., Jiang G., Zhou Y., Liu Q., Su Q., Wei W., Du J., Wang H. (2017). Activation of GPER suppresses migration and angiogenesis of triple negative breast cancer via inhibition of NF-κB/IL-6 signals. Cancer Lett..

[117] Ma D., Panda S., Lin J.D. (2011). Temporal orchestration of circadian autophagy rhythm by C/EBPβ. EMBO J..

[118] Torres-Avilés N.A., Albores-García D., Luna A.L., Moreno-Galván M., Salgado-Bustamante M., Portales-Pérez D.P., Calderón-Aranda E.S. (2016). Exposure to *p*,*p*’-DDE Induces Morphological Changes and Activation of the PKC*α*-p38-C/EBP*β* Pathway in Human Promyelocytic HL-60 Cells. Biomed. Res. Int..

[119] Lahn M., Köhler G., Sundell K., Su C., Li S., Paterson B.M., Bumol T.F. (2004). Protein kinase C alpha expression in breast and ovarian cancer. Oncology.

[120] Khristi V., Chakravarthi V.P., Singh P., Ghosh S., Pramanik A., Ratri A., Borosha S., Roby K.F., Wolfe M.W., Rumi M.A.K. (2018). ESR2 regulates granulosa cell genes essential for follicle maturation and ovulation. Mol. Cell. Endocrinol..

[121] Niehof M., Manns M.P., Trautwein C. (1997). CREB controls LAP/C/EBP beta transcription. Mol. Cell. Biol..

[122] Yu T., Yang G., Hou Y., Tang X., Wu C., Wu X.A., Guo L., Zhu Q., Luo H., Du Y.E. (2017). Cytoplasmic GPER translocation in cancer-associated fibroblasts mediates cAMP/PKA/CREB/glycolytic axis to confer tumor cells with multidrug resistance. Oncogene.

[123] Sanchez A.M., Csibi A., Raibon A., Cornille K., Gay S., Bernardi H., Candau R. (2012). AMPK promotes skeletal muscle autophagy through activation of forkhead FoxO3a and interaction with Ulk1. J. Cell. Biochem..

[124] Audesse A.J., Dhakal S., Hassell L.A., Gardell Z., Nemtsova Y., Webb A.E. (2019). FOXO3 directly regulates an autophagy network to functionally regulate proteostasis in adult neural stem cells. PLoS Genet..

[125] Sisci D., Maris P., Cesario M.G., Anselmo W., Coroniti R., Trombino G.E., Romeo F., Ferraro A., Lanzino M., Aquila S. (2013). The estrogen receptor α is the key regulator of the bifunctional role of FoxO3a transcription factor in breast cancer motility and invasiveness. Cell Cycle.

[126] Thompson M.G., Peiffer D.S., Larson M., Navarro F., Watkins S.K. (2017). FOXO3, estrogen receptor alpha, and androgen receptor impact tumor growth rate and infiltration of dendritic cell subsets differentially between male and female mice. Cancer Immunol. Immunother..

[127] Zekas E., Prossnitz E.R. (2015). Estrogen-mediated inactivation of FOXO3a by the G protein-coupled estrogen receptor GPER. BMC Cancer.

[128] Zhang T., Liu J., Shen S., Tong Q., Ma X., Lin L. (2020). SIRT3 promotes lipophagy and chaperon-mediated autophagy to protect hepatocytes against lipotoxicity. Cell Death Differ..

[129] Panza S., Santoro M., De Amicis F., Morelli C., Passarelli V., D’Aquila P., Giordano F., Cione E., Passarino G., Bellizzi D. (2017). Estradiol via estrogen receptor beta influences ROS levels through the transcriptional regulation of SIRT3 in human seminoma TCam-2 cells. Tumour Biol..

[130] Goldberg A.A., Nkengfac B., Sanchez A.M.J., Moroz N., Qureshi S.T., Koromilas A.E., Wang S., Burelle Y., Hussain S.N., Kristof A.S. (2017). Regulation of ULK1 Expression and Autophagy by STAT1. J. Biol. Chem..

[131] Young N.A., Valiente G.R., Hampton J.M., Wu L.C., Burd C.J., Willis W.L., Bruss M., Steigelman H., Gotsatsenko M., Amici S.A. (2017). Estrogen-regulated STAT1 activation promotes TLR8 expression to facilitate signaling via microRNA-21 in systemic lupus erythematosus. Clin. Immunol..

[132] Kenzelmann Broz D., Spano Mello S., Bieging K.T., Jiang D., Dusek R.L., Brady C.A., Sidow A., Attardi L.D. (2013). Global genomic profiling reveals an extensive p53-regulated autophagy program contributing to key p53 responses. Genes. Dev..

[133] Konduri S.D., Medisetty R., Liu W., Kaipparettu B.A., Srivastava P., Brauch H., Fritz P., Swetzig W.M., Gardner A.E., Khan S.A. (2010). Mechanisms of estrogen receptor antagonism toward p53 and its implications in breast cancer therapeutic response and stem cell regulation. Proc. Natl. Acad. Sci. USA.

[134] Lu W., Katzenellenbogen B.S. (2017). Estrogen Receptor-β Modulation of the ERα-p53 Loop Regulating Gene Expression, Proliferation, and Apoptosis in Breast Cancer. Horm. Cancer.

[135] Chauhan S., Goodwin J.G., Chauhan S., Manyam G., Wang J., Kamat A.M., Boyd D.D. (2013). ZKSCAN3 is a master transcriptional repressor of autophagy. Mol. Cell.

[136] Li Y., Xu M., Ding X., Yan C., Song Z., Chen L., Huang X., Wang X., Jian Y., Tang G. (2016). Protein kinase C controls lysosome biogenesis independently of mTORC1. Nat. Cell Biol..

[137] Xiong X., Tao R., DePinho R.A., Dong X.C. (2012). The autophagy-related gene 14 (Atg14) is regulated by forkhead box O transcription factors and circadian rhythms and plays a critical role in hepatic autophagy and lipid metabolism. J. Biol. Chem..

[138] Tamatani M., Che Y.H., Matsuzaki H., Ogawa S., Okado H., Miyake S., Mizuno T., Tohyama M. (1999). Tumor necrosis factor induces Bcl-2 and Bcl-x expression through NFkappaB activation in primary hippocampal neurons. J. Biol. Chem..

[139] Settembre C., De Cegli R., Mansueto G., Saha P.K., Vetrini F., Visvikis O., Huynh T., Carissimo A., Palmer D., Klisch T.J. (2013). TFEB controls cellular lipid metabolism through a starvation-induced autoregulatory loop. Nat. Cell Biol..

[140] Napolitano G., Ballabio A. (2016). TFEB at a glance. J. Cell Sci..

[141] Peña-Llopis S., Vega-Rubin-de-Celis S., Schwartz J.C., Wolff N.C., Tran T.A., Zou L., Xie X.J., Corey D.R., Brugarolas J. (2011). Regulation of TFEB and V-ATPases by mTORC1. EMBO J..

[142] Kang J., Chong S.J., Ooi V.Z., Vali S., Kumar A., Kapoor S., Abbasi T., Hirpara J.L., Loh T., Goh B.C. (2015). Overexpression of Bcl-2 induces STAT-3 activation via an increase in mitochondrial superoxide. Oncotarget.

[143] Binai N.A., Damert A., Carra G., Steckelbroeck S., Löwer J., Löwer R., Wessler S. (2010). Expression of estrogen receptor alpha increases leptin-induced STAT3 activity in breast cancer cells. Int. J. Cancer.

[144] Ge Q., Lu M., Ju L., Qian K., Wang G., Wu C.L., Liu X., Xiao Y., Wang X. (2019). miR-4324-RACGAP1-STAT3-ESR1 feedback loop inhibits proliferation and metastasis of bladder cancer. Int. J. Cancer.

[145] Scherz-Shouval R., Weidberg H., Gonen C., Wilder S., Elazar Z., Oren M. (2010). p53-dependent regulation of autophagy protein LC3 supports cancer cell survival under prolonged starvation. Proc. Natl. Acad. Sci. USA.

[146] Ahmed M., Lai T.H., Hwang J.S., Zada S., Pham T.M., Kim D.R. (2019). Transcriptional Regulation of Autophagy Genes via Stage-Specific Activation of CEBPB and PPARG during Adipogenesis: A Systematic Study Using Public Gene Expression and Transcription Factor Binding Datasets. Cells.

[147] Webb A.E., Brunet A. (2014). FOXO transcription factors: Key regulators of cellular quality control. Trends Biochem. Sci..

[148] Li D.D., Wang L.L., Deng R., Tang J., Shen Y., Guo J.F., Wang Y., Xia L.P., Feng G.K., Liu Q.Q. (2009). The pivotal role of c-Jun NH2-terminal kinase-mediated Beclin 1 expression during anticancer agents-induced autophagy in cancer cells. Oncogene.

[149] Chimento A., Sirianni R., Casaburi I., Ruggiero C., Maggiolini M., Andò S., Pezzi V. (2012). 17β-Estradiol activates GPER- and ESR1-dependent pathways inducing apoptosis in GC-2 cells, a mouse spermatocyte-derived cell line. Mol. Cell. Endocrinol..

[150] De Francesco E.M., Angelone T., Pasqua T., Pupo M., Cerra M.C., Maggiolini M. (2013). GPER mediates cardiotropic effects in spontaneously hypertensive rat hearts. PLoS ONE.

[151] Copetti T., Bertoli C., Dalla E., Demarchi F., Schneider C. (2009). p65/RelA modulates BECN1 transcription and autophagy. Mol. Cell. Biol..

[152] Zhang Y.G., Zhu X., Lu R., Messer J.S., Xia Y., Chang E.B., Sun J. (2019). Intestinal epithelial HMGB1 inhibits bacterial infection via STAT3 regulation of autophagy. Autophagy.

[153] Mammucari C., Milan G., Romanello V., Masiero E., Rudolf R., Del Piccolo P., Burden S.J., Di Lisi R., Sandri C., Zhao J. (2007). FoxO3 controls autophagy in skeletal muscle in vivo. Cell Metab..

[154] Lee J.W., Nam H., Kim L.E., Jeon Y., Min H., Ha S., Lee Y., Kim S.Y., Lee S.J., Kim E.K. (2019). TLR4 (toll-like receptor 4) activation suppresses autophagy through inhibition of FOXO3 and impairs phagocytic capacity of microglia. Autophagy.

[155] Sutton M.N., Yang H., Huang G.Y., Fu C., Pontikos M., Wang Y., Mao W., Pang L., Yang M., Liu J. (2018). RAS-related GTPases DIRAS1 and DIRAS2 induce autophagic cancer cell death and are required for autophagy in murine ovarian cancer cells. Autophagy.

[156] Fitzwalter B.E., Towers C.G., Sullivan K.D., Andrysik Z., Hoh M., Ludwig M., O’Prey J., Ryan K.M., Espinosa J.M., Morgan M.J. (2018). Autophagy Inhibition Mediates Apoptosis Sensitization in Cancer Therapy by Relieving FOXO3a Turnover. Dev. Cell.

[157] Liu P., Huang G., Wei T., Gao J., Huang C., Sun M., Zhu L., Shen W. (2018). Sirtuin 3-induced macrophage autophagy in regulating NLRP3 inflammasome activation. Biochim. Biophys. Acta Mol. Basis Dis..

[158] Schips T.G., Wietelmann A., Höhn K., Schimanski S., Walther P., Braun T., Wirth T., Maier H.J. (2011). FoxO3 induces reversible cardiac atrophy and autophagy in a transgenic mouse model. Cardiovasc. Res..

[159] Shaw J., Yurkova N., Zhang T., Gang H., Aguilar F., Weidman D., Scramstad C., Weisman H., Kirshenbaum L.A. (2008). Antagonism of E2F-1 regulated Bnip3 transcription by NF-kappaB is essential for basal cell survival. Proc. Natl. Acad. Sci. USA.

[160] Yang J., AlTahan A., Jones D.T., Buffa F.M., Bridges E., Interiano R.B., Qu C., Vogt N., Li J.L., Baban D. (2015). Estrogen receptor-α directly regulates the hypoxia-inducible factor 1 pathway associated with antiestrogen response in breast cancer. Proc. Natl. Acad. Sci. USA.

[161] De Francesco E.M., Pellegrino M., Santolla M.F., Lappano R., Ricchio E., Abonante S., Maggiolini M. (2014). GPER mediates activation of HIF1α/VEGF signaling by estrogens. Cancer Res..

[162] Lu D., Qu Y., Shi F., Feng D., Tao K., Gao G., He S., Zhao T. (2016). Activation of G protein-coupled estrogen receptor 1 (GPER-1) ameliorates blood-brain barrier permeability after global cerebral ischemia in ovariectomized rats. Biochem. Biophys. Res. Commun..

[163] Pratt J., Annabi B. (2014). Induction of autophagy biomarker BNIP3 requires a JAK2/STAT3 and MT1-MMP signaling interplay in Concanavalin-A-activated U87 glioblastoma cells. Cell. Signal.

[164] Sun T., Li D., Wang L., Xia L., Ma J., Guan Z., Feng G., Zhu X. (2011). c-Jun NH2-terminal kinase activation is essential for up-regulation of LC3 during ceramide-induced autophagy in human nasopharyngeal carcinoma cells. J. Transl. Med..

[165] Petherick K.J., Williams A.C., Lane J.D., Ordóñez-Morán P., Huelsken J., Collard T.J., Smartt H.J., Batson J., Malik K., Paraskeva C. (2013). Autolysosomal β-catenin degradation regulates Wnt-autophagy-p62 crosstalk. EMBO J..

[166] Kouzmenko A.P., Takeyama K., Ito S., Furutani T., Sawatsubashi S., Maki A., Suzuki E., Kawasaki Y., Akiyama T., Tabata T. (2004). Wnt/beta-catenin and estrogen signaling converge in vivo. J. Biol. Chem..

[167] Duran A., Linares J.F., Galvez A.S., Wikenheiser K., Flores J.M., Diaz-Meco M.T., Moscat J. (2008). The signaling adaptor p62 is an important NF-kappaB mediator in tumorigenesis. Cancer Cell.

[168] Xiang X., Huang J., Song S., Wang Y., Zeng Y., Wu S., Ruan Y. (2020). 17β-estradiol inhibits H_2_O_2_-induced senescence in HUVEC cells through upregulating SIRT3 expression and promoting autophagy. Biogerontology.

[169] Song H., Feng X., Zhang H., Luo Y., Huang J., Lin M., Jin J., Ding X., Wu S., Huang H. (2019). METTL3 and ALKBH5 oppositely regulate m^6^A modification of *TFEB* mRNA, which dictates the fate of hypoxia/reoxygenation-treated cardiomyocytes. Autophagy.

[170] Li S., Song Y., Quach C., Guo H., Jang G.B., Maazi H., Zhao S., Sands N.A., Liu Q., In G.K. (2019). Transcriptional regulation of autophagy-lysosomal function in BRAF-driven melanoma progression and chemoresistance. Nat. Commun..

[171] Molloy T.J., Vu T.T., Stölzel F., Ehninger G., Ma D. (2016). Mir-10a Regulates Autophagy and Cell Cycle in Normal Karyotype Acute Myeloid Leukaemia. Blood.

[172] Hara T., Takamura A., Kishi C., Iemura S., Natsume T., Guan J.L., Mizushima N. (2008). FIP200, a ULK-interacting protein, is required for autophagosome formation in mammalian cells. J. Cell Biol..

[173] Zhang J., Ren L., Yu M., Liu X., Ma W., Huang L., Li X., Ye X. (2019). S-equol inhibits proliferation and promotes apoptosis of human breast cancer MCF-7 cells via regulating miR-10a-5p and PI3K/AKT pathway. Arch. Biochem. Biophys..

[174] Wu H., Wang F., Hu S., Yin C., Li X., Zhao S., Wang J., Yan X. (2012). MiR-20a and miR-106b negatively regulate autophagy induced by leucine deprivation via suppression of ULK1 expression in C2C12 myoblasts. Cell. Signal.

[175] Castellano L., Giamas G., Jacob J., Coombes R.C., Lucchesi W., Thiruchelvam P., Barton G., Jiao L.R., Wait R., Waxman J. (2009). The estrogen receptor-alpha-induced microRNA signature regulates itself and its transcriptional response. Proc. Natl. Acad. Sci. USA.

[176] Williams K.C., Renthal N.E., Gerard R.D., Mendelson C.R. (2012). The microRNA (miR)-199a/214 cluster mediates opposing effects of progesterone and estrogen on uterine contractility during pregnancy and labor. Mol. Endocrinol..

[177] Zhou J., Yao W., Liu K., Wen Q., Wu W., Liu H., Li Q. (2016). MicroRNA let-7g regulates mouse granulosa cell autophagy by targeting insulin-like growth factor 1 receptor. Int. J. Biochem. Cell Biol..

[178] Klinge C.M. (2012). miRNAs and estrogen action. Trends Endocrinol. Metab..

[179] Qian P., Zuo Z., Wu Z., Meng X., Li G., Wu Z., Zhang W., Tan S., Pandey V., Yao Y. (2011). Pivotal role of reduced let-7g expression in breast cancer invasion and metastasis. Cancer Res..

[180] Sims E.K., Lakhter A.J., Anderson-Baucum E., Kono T., Tong X., Evans-Molina C. (2017). MicroRNA 21 targets BCL2 mRNA to increase apoptosis in rat and human beta cells. Diabetologia.

[181] Teng Y., Litchfield L.M., Ivanova M.M., Prough R.A., Clark B.J., Klinge C.M. (2014). Dehydroepiandrosterone-induces miR-21 transcription in HepG2 cells through estrogen receptor β and androgen receptor. Mol. Cell. Endocrinol..

[182] Lu M., Qin X., Zhou Y., Li G., Liu Z., Yue H., Geng X. (2020). LncRNA HOTAIR suppresses cell apoptosis, autophagy and induces cell proliferation in cholangiocarcinoma by modulating the miR-204-5p/HMGB1 axis. Biomed. Pharmacother..

[183] Tao S., He H., Chen Q. (2015). Estradiol induces HOTAIR levels via GPER-mediated miR-148a inhibition in breast cancer. J. Transl. Med..

[184] McCormick J., Suleman N., Scarabelli T.M., Knight R.A., Latchman D.S., Stephanou A. (2012). STAT1 deficiency in the heart protects against myocardial infarction by enhancing autophagy. J. Cell. Mol. Med..

[185] Li D., Li C., Chen Y., Teng L., Cao Y., Wang W., Pan H., Xu Y., Yang D. (2020). LncRNA HOTAIR induces sunitinib resistance in renal cancer by acting as a competing endogenous RNA to regulate autophagy of renal cells. Cancer Cell Int..

[186] Yang L., Zhang X., Li H., Liu J. (2016). The long noncoding RNA HOTAIR activates autophagy by upregulating ATG3 and ATG7 in hepatocellular carcinoma. Mol. Biosyst..

[187] Comincini S., Allavena G., Palumbo S., Morini M., Durando F., Angeletti F., Pirtoli L., Miracco C. (2013). microRNA-17 regulates the expression of ATG7 and modulates the autophagy process, improving the sensitivity to temozolomide and low-dose ionizing radiation treatments in human glioblastoma cells. Cancer Biol. Ther..

[188] Wu C., Yang L., Qi X., Wang T., Li M., Xu K. (2018). Inhibition of long non-coding RNA HOTAIR enhances radiosensitivity via regulating autophagy in pancreatic cancer. Cancer Manag. Res..

[189] Lang Y., Li Y., Yu H., Lin L., Chen X., Wang S., Zhang H. (2020). HOTAIR drives autophagy in midbrain dopaminergic neurons in the substantia nigra compacta in a mouse model of Parkinson’s disease by elevating NPTX2 *via* miR-221-3p binding. Aging.

[190] Diano S., Horvath T.L. (2012). Mitochondrial uncoupling protein 2 (UCP2) in glucose and lipid metabolism. Trends Mol. Med..

[191] Yu G., Wang J., Xu K., Dong J. (2016). Dynamic regulation of uncoupling protein 2 expression by microRNA-214 in hepatocellular carcinoma. Biosci. Rep..

[192] Yu Y., Zhang X., Tian H., Zhang Z., Tian Y. (2018). Knockdown of long non-coding RNA HOTAIR increases cisplatin sensitivity in ovarian cancer by inhibiting cisplatin-induced autophagy. J. BUON.

[193] Wang X., Cheng M.L., Gong Y., Ma W.J., Li B., Jiang Y.Z. (2020). LncRNA DANCR promotes ATG7 expression to accelerate hepatocellular carcinoma cell proliferation and autophagy by sponging miR-222-3p. Eur. Rev. Med. Pharmacol. Sci..

[194] Mozdarani H., Ezzatizadeh V., Rahbar Parvaneh R. (2020). The emerging role of the long non-coding RNA HOTAIR in breast cancer development and treatment. J. Transl. Med..

[195] Zhang Y., Ai H., Fan X., Chen S., Wang Y., Liu L. (2020). Knockdown of long non-coding RNA HOTAIR reverses cisplatin resistance of ovarian cancer cells through inhibiting miR-138-5p-regulated EZH2 and SIRT1. Biol. Res..

[196] Tang B., Bao N., He G., Wang J. (2019). Long noncoding RNA HOTAIR regulates autophagy via the miR-20b-5p/ATG7 axis in hepatic ischemia/reperfusion injury. Gene.

[197] Yang L., Peng X., Li Y., Zhang X., Ma Y., Wu C., Fan Q., Wei S., Li H., Liu J. (2019). Long non-coding RNA HOTAIR promotes exosome secretion by regulating RAB35 and SNAP23 in hepatocellular carcinoma. Mol. Cancer.

[198] Pawłowska E., Szczepanska J., Blasiak J. (2017). The Long Noncoding RNA HOTAIR in Breast Cancer: Does Autophagy Play a Role?. Int. J. Mol. Sci..

[199] Imesch P., Samartzis E.P., Dedes K.J., Fink D., Fedier A., Imesch P., Samartzis E.P., Dedes K.J., Fink D., Fedier A. (2013). Histone deacetylase inhibitors down-regulate G-protein-coupled estrogen receptor and the GPER-antagonist G-15 inhibits proliferation in endometriotic cells. Fertil. Steril..

[200] Fatoba S.T., Okorokov A.L. (2011). Human SIRT1 associates with mitotic chromatin and contributes to chromosomal condensation. Cell Cycle.

[201] Vazquez M.J., Toro C.A., Castellano J.M., Ruiz-Pino F., Roa J., Beiroa D., Heras V., Velasco I., Dieguez C., Pinilla L. (2018). SIRT1 mediates obesity- and nutrient-dependent perturbation of pubertal timing by epigenetically controlling Kiss1 expression. Nat. Commun..

[202] Santolla M.F., Avino S., Pellegrino M., De Francesco E.M., De Marco P., Lappano R., Vivacqua A., Cirillo F., Rigiracciolo D.C., Scarpelli A. (2015). SIRT1 is involved in oncogenic signaling mediated by GPER in breast cancer. Cell Death Dis..

[203] Holloway K.R., Barbieri A., Malyarchuk S., Saxena M., Nedeljkovic-Kurepa A., Cameron Mehl M., Wang A., Gu X., Pruitt K. (2013). SIRT1 positively regulates breast cancer associated human aromatase (CYP19A1) expression. Mol. Endocrinol..

[204] Molehin D., Castro-Piedras I., Sharma M., Sennoune S.R., Arena D., Manna P.R., Pruitt K. (2018). Aromatase Acetylation Patterns and Altered Activity in Response to Sirtuin Inhibition. Mol. Cancer Res..

[205] Wei S., Liu W., Sun N., Wu Y., Song H., Wang C., Wang S., Zou R., Lin L., Zeng K. (2021). MOF upregulates the estrogen receptor α signaling pathway by its acetylase activity in hepatocellular carcinoma. Cancer Sci..

[206] Bannister A.J., Kouzarides T. (2011). Regulation of chromatin by histone modifications. Cell Res..

[207] Duteil D., Tourrette Y., Eberlin A., Willmann D., Patel D., Friedrichs N., Müller J.M., Schüle R. (2018). The histone acetyltransferase inhibitor Nir regulates epidermis development. Development.

[208] Sharma S., Sarathlal K.C., Taliyan R. (2019). Epigenetics in Neurodegenerative Diseases: The Role of Histone Deacetylases. CNS Neurol. Disord. Drug Targets.

[209] Col E., Caron C., Seigneurin-Berny D., Gracia J., Favier A., Khochbin S. (2001). The histone acetyltransferase, hGCN5, interacts with and acetylates the HIV transactivator, Tat. J. Biol. Chem..

[210] Zhang Q., Yao H., Vo N., Goodman R.H. (2000). Acetylation of adenovirus E1A regulates binding of the transcriptional corepressor CtBP. Proc. Natl. Acad. Sci. USA.

[211] González-Rojo S., Lombó M., Fernández-Díez C., Herráez M.P. (2019). Male exposure to bisphenol a impairs spermatogenesis and triggers histone hyperacetylation in zebrafish testes. Environ. Pollut..

[212] Gao Y., Chen L., Han Y., Wu F., Yang W.S., Zhang Z., Huo T., Zhu Y., Yu C., Kim H. (2020). Acetylation of histone H3K27 signals the transcriptional elongation for estrogen receptor alpha. Commun. Biol..

[213] Han N., Heublein S., Jeschke U., Kuhn C., Hester A., Czogalla B., Mahner S., Rottmann M., Mayr D., Schmoeckel E. (2021). The G-Protein-Coupled Estrogen Receptor (GPER) Regulates Trimethylation of Histone H3 at Lysine 4 and Represses Migration and Proliferation of Ovarian Cancer Cells In Vitro. Cells.

[214] Khan M., Ullah R., Rehman S.U., Shah S.A., Saeed K., Muhammad T., Park H.Y., Jo M.H., Choe K., Rutten B.P.F. (2019). 17β-Estradiol Modulates SIRT1 and Halts Oxidative Stress-Mediated Cognitive Impairment in a Male Aging Mouse Model. Cells.

[215] Li H., Xia N., Hasselwander S., Daiber A.A. (2019). Resveratrol and Vascular Function. Int. J. Mol. Sci..

[216] Dai R., Ahmed S.A. (2014). Sexual dimorphism of miRNA expression: A new perspective in understanding the sex bias of autoimmune diseases. Ther. Clin. Risk Manag..

[217] Martinez-Outschoorn U.E., Pavlides S., Whitaker-Menezes D., Daumer K.M., Milliman J.N., Chiavarina B., Migneco G., Witkiewicz A.K., Martinez-Cantarin M.P., Flomenberg N. (2010). Tumor cells induce the cancer associated fibroblast phenotype via caveolin-1 degradation: Implications for breast cancer and DCIS therapy with autophagy inhibitors. Cell Cycle.

[218] Massarweh S., Romond E., Black E.P., Van Meter E., Shelton B., Kadamyan-Melkumian V., Stevens M., Elledge R. (2014). A phase II study of combined fulvestrant and everolimus in patients with metastatic estrogen receptor (ER)-positive breast cancer after aromatase inhibitor (AI) failure. Breast Cancer Res. Treat..

[219] Zhao Y., Wang X., Liu Y., Wang H.Y., Xiang J. (2022). The effects of estrogen on targeted cancer therapy drugs. Pharmacol. Res..

[220] Belchetz P.E. (1994). Hormonal treatment of postmenopausal women. N. Engl. J. Med..

[221] Lewis J.S., Osipo C., Meeke K., Jordan V.C. (2005). Estrogen-induced apoptosis in a breast cancer model resistant to long-term estrogen withdrawal. J. Steroid Biochem. Mol. Biol..

[222] Suba Z. (2013). Circulatory estrogen level protects against breast cancer in obese women. Recent Pat. Anticancer Drug Discov..

[223] Chakravarty D., Nair S.S., Hammouda N., Ratnani P., Gharib Y., Wagaskar V., Mohamed N., Lundon D., Dovey Z., Kyprianou N. (2020). Sex differences in SARS-CoV-2 infection rates and the potential link to prostate cancer. Commun. Biol..

[224] Nicastri E., Marinangeli F., Pivetta E., Torri E., Reggiani F., Fiorentino G., Scorzolini L., Vettori S., Marsiglia C., Gavioli E.M. (2022). A phase 2 randomized, double-blinded, placebo-controlled, multicenter trial evaluating the efficacy and safety of raloxifene for patients with mild to moderate COVID-19. EClinicalMedicine.

[225] Ra R., Kim J.S., Jeong K.H., Hwang H.S. (2021). COVID-19 and Sirolimus Treatment in a Kidney Transplant Recipient. Exp. Clin. Transplant..

[226] Réa-Neto Á., Bernardelli R.S., Câmara B.M.D., Reese F.B., Queiroga M.V.O., Oliveira M.C. (2021). An open-label randomized controlled trial evaluating the efficacy of chloroquine/hydroxychloroquine in severe COVID-19 patients. Sci. Rep..

[227] Wang C.H., Chung F.T., Lin S.M., Huang S.Y., Chou C.L., Lee K.Y., Lin T.Y., Kuo H.P. (2014). Adjuvant treatment with a mammalian target of rapamycin inhibitor, sirolimus, and steroids improves outcomes in patients with severe H1N1 pneumonia and acute respiratory failure. Crit. Care Med..

[228] Zurlo M., Nicoli F., Borgatti M., Finotti A., Gambari R. (2022). Possible effects of sirolimus treatment on the long-term efficacy of COVID-19 vaccination in patients with β-thalassemia: A theoretical perspective. Int. J. Mol. Med..

[229] Fatima L.A., Campello R.S., Santos R.S., Freitas H.S., Frank A.P., Machado U.F., Clegg D.J. (2017). Estrogen receptor 1 (ESR1) regulates VEGFA in adipose tissue. Sci. Rep..

[230] Xia X., Zhou C., He X., Liu C., Wang G., Sun X. (2020). The relationship between estrogen-induced phenotypic transformation and proliferation of vascular smooth muscle and hypertensive intracerebral hemorrhage. Ann. Transl. Med..

[231] Toriola A.T., Vääräsmäki M., Lehtinen M., Zeleniuch-Jacquotte A., Lundin E., Rodgers K.G., Lakso H.A., Chen T., Schock H., Hallmans G. (2011). Determinants of maternal sex steroids during the first half of pregnancy. Obstet. Gynecol..

[232] Kiesner J. (2017). The Menstrual Cycle-Response and Developmental Affective-Risk Model: A multilevel and integrative model of influence. Psychol. Rev..

[233] Ghosh M., Gälman C., Rudling M., Angelin B. (2015). Influence of physiological changes in endogenous estrogen on circulating PCSK9 and LDL cholesterol. J. Lipid Res..

[234] Savolainen-Peltonen H., Vihma V., Wang F., Turpeinen U., Hämäläinen E., Haanpää M., Leidenius M., Tikkanen M.J., Mikkola T.S. (2018). Estrogen biosynthesis in breast adipose tissue during menstrual cycle in women with and without breast cancer. Gynecol. Endocrinol..

[235] Gałązka A., Jankiewicz U. (2022). Endocrine Disrupting Compounds (Nonylphenol and Bisphenol A)-Sources, Harmfulness and Laccase-Assisted Degradation in the Aquatic Environment. Microorganisms.

