# Supplementary material for: The Estrogen–Autophagy Axis: Insights into Cytoprotection and Therapeutic Potential in Cancer and Infection
Source: Int J Mol Sci. 2024 Nov 22;25(23):12576. doi: 10.3390/ijms252312576 (PMC11641569; doi:10.3390/ijms252312576)
Supplement: Supplementary file 1 [file ijms-25-12576-s001.zip › ijms-3302700-supplementary.pdf]

International Journal of
Molecular Sciences

Table S1. E2 regulation of autophagy.

Ligand	Action on autophagy	Disease	Tissue	Cell Line or Animal	Dose	Effector proteins	Autophagy morphology	Ref
E2	Induce	Breast cancer	breast	MCF-7, MDA-MB-231	10, 50 nM	MAP1LC3-II ↓, BCL2, BECN1, CLDN6, CCND1, DNM2, ESR1 ↓, ESR2, IGF1R, SQSTM1, p-AKT1	autophagosome	[27], [28]
		cardiovascular diseases	cardiovascular	HUVEC	0.1 μM	MAP1LC3-II/ MAP1LC3-I, BECN1, EDN1 ↓, TP53, SQSTM1 ↓, p-TP53, NO, BBC3/PUMA, p-RB1 ↓	autophagosome	[14]
		Hypoxic pulmonary hypertension	lung	PAEC	1, 10 nM	MAP1LC3-II, ESR-independent, MAPK1 ↓	NA	[31]
				male Sprague-Dawley rats exposed to hypobaric hypoxia	75 μg/kg/day	MAP1LC3-II, MAP1LC3-I, IFI27, MAPK1/3 ↓, **VEGF ↓**	NA	[30]
		Intervertebral disc degeneration	bone	nucleus pulposus cells	10^{-11}, 10^{-9}, 10^{-7} M	MAP1LC3-II ↑, MAP1LC3-I ↓, BECN1, **MMP3 ↓**, **MMP13 ↓**, **cleaved CASP3 ↓**	NA	[32]
		Osteocyte survival	bone	female Wistar rats	SHAM, OVX, diethylstilbestrol, 30 μg/kg	MAP1LC3-II/MAP1LC3-I, SQSTM1 ↓, BECN1	NA	[33]
		Osteoporosis	bone	murine osteoblastic cell line MC3T3-E1	10^{-9}, 10^{-7}M	MAP1LC3-II/ MAP1LC3-I, BCL2 ↓, BECN1, ULK1, GPER1, p-AKT1, p-MTOR ↓, p-HSPD1/HSP60, p-MAPK1-MAPK3, **cleaved CASP3 ↓**	lysosome, autolysosome, mitochondrial autophagosome	[36], [18]
			bone	human primary mesenchymal stem cells	1 nM	MAP1LC3-II, ATG16L1, RAB3GAP1, SQSTM1 ↓	NA	[34]
		Parkinson disease	CNS	/	1 mg/kg/day	MAP1LC3-II, MAPK1	autolysosomes/ autophagosome	[37]
		Renal cell carcinoma	kidney	RCC cell lines	7, 28 μM	MAP1LC3B-II, MAP1LC3B-I ↓, SQSTM1	autophagosome	[29]
		Retinal degeneration	eye	female SD rats	4 μL of 10^{-5} M	MAP1LC3-II/MAP1LC3-I, BECN1	autophagosome	[35]
		Testicular germ cell tumors	testis	human testicular cancer TCam-2	1, 10, 100 nM	BECN1, AMBRA1, PIK3C3, UVRAG, PIK3CA-III, ESR2 ↓, AKT1 ↓	autophagic vesicle	[19]
	restrict (prevent induced autophagy)	Endometriosis	uterus	endometrial stromal cells	10^{-8} M	MAP1LC3B-II, MAP1LC3B-I ↓, BECN1 ↓,	NA	[20]
		Heart diseases	heart	rat cardiomyocyte H9c2	10 nM	MAP1LC3-II ↓, MAP1LC3-I, BCL2 ↓, ESR1, ESR2, BNIP3 ↓, AKT1, ATG5 ↓, BECN1 ↓, HIF1A ↓, **IGFBP3 ↓**	autophagosome	[30], [237]
		Hepatocellular carcinoma	liver	human liver carcinoma HepG2	50, 100 nM	MAP1LC3B-II/ MAP1LC3B-I ↓, SQSTM1, AMPK ↓, BECN1 ↓, MTOR, **NLRP3 inflammasome**	autophagic vacuole	[21]
		Intervertebral disc degeneration	bone	Sprague-Dawley rats	10 μg/kg/day	MAP1LC3-II ↓, MAP1LC3-I ↓, BECN1 ↓, ATG5 ↓	autophagosome	[22]

		Ischemic brain injury	brain	C57BL/6 mice	0.05 mg	MAP1LC3-II↓, MAP1LC3-I↓, SQSTM1, BECN1↓, ATG5↓	NA	[23]
		Myocardial injury	heart	cardiomyocytes	10 nM	MAP1LC3-II/ MAP1LC3-I, ATG5↓, BECN1↓	NA	[40]
		NaAsO2-induced nephrotoxicity	proximal renal tubule	murine renal proximal tubular epithelial cell line mProx24	0.2 mg/kg/day	MAP1LC3-II↑, MAP1LC3-I↓, SQSTM1↑, SOCS3, STAT3↓, MAPK↓	autophagosome	[24]
		Ovariectomy	proximal tibias	/	10 μg/kg/day	MAP1LC3-II↓, MAP1LC3-I↓, SQSTM1, ATG5↓, BECN1↓	acidic vesicular	[41]
		Spinal cord injury	adrenal medulla	rat pheochromocytoma PC12	20 nM	MAP1LC3-II↓, MAP1LC3-I, SQSTM1, BECN1↓, ATG5↓, ATG7↓	NA	[39]
		Visceral adiposity	visceral fat	murine embryonic fibroblasts/preadipocytes 3T3L1	0.1 μM/day	MAP1LC3-II, SQSTM1, MTOR, ULK1	NA	[25]

Abbreviations: AKT1/PKB, AKT serine/threonine kinase 1; AMBRA1, autophagy and beclin 1 regulator 1; ATG, autophagy related; ATG16L1, autophagy related 16 like 1; BBC3/PUMA, Bcl-2 binding component 3 in mouse and p53 upregulated modulator of apoptosis in human; BECN1, beclin 1; BNIP3, BCL2 interacting protein 3; CCND1, cyclin D1; CNS, central nervous system; E2, 17β-estradiol; EDN1, endothelin 1; GAPDH, glyceraldehyde-3-phosphate dehydrogenase; HUVECs, human umbilical vein endothelial cells; IFI27/p27, interferon alpha inducible protein 27; IGF1R, insulin like growth factor 1 receptor; IGFBP3, insulin like growth factor binding protein 3; MAP1LC3/LC3, microtubule associated protein 1 light chain 3; MAPK1/ERK2, mitogen-activated protein kinase 1; MAPK3/ERK1, mitogen-activated protein kinase 3; PAEC, pulmonary artery endothelial cells; PIK3C3/VPS34, phosphatidylinositol 3-kinase catalytic subunit type 3; PLIN2/ADRP, perilipin 2; RAB3GAP1, RAB3 GTPase activating protein catalytic subunit 1; RB1, RB transcriptional corepressor 1; SOCS3, suppressor of cytokine signaling 3; SQSTM1/p62, sequestosome 1; STAT, signal transducer and activator of transcription; TP53, tumor protein p53; ULK1, unc-51 like autophagy activating kinase 1; UVRAG, UV radiation resistance associated; VEGF, vascular endothelial growth factor; ↓, E2-induced downregulation; no arrow, E2-induced upregulation; bold font, effector proteins associated with other reactions in cells, such as inflammation, proliferation, migration, apoptosis, and immunity.

Table S2. Regulation of autophagy by ESR ligands.

**Type**	**Ligand**	**Disease**	**Tissue**	**Cell line**	**Dose**	**Effector proteins**	**Autophagy morphology**	**Ref**
**ESR1 antagonist and GPER agonist**	4-OH TAM	Breast cancer	breast	MCF-7, T47D, BT-474, tam	0.5, 1, 5 μM	MAP1LC3-II, NO, MAPK1, PLIN2, BCL2↓	autophagosome, autolysosome	[43, 44]
	fulvestrant	Breast cancer	breast	MCF-7	1 μM	MAP1LC3-II, MAP1LC3-I, BCL2↓, BECN1, AKT1↓, MAPK/ERK	autophagosome	[46, 56]
		cardiovascular diseases (H_2O_2-induced + E2)	cardiovascular	HUVEC	0.1 μM	MAP1LC3-II/ MAP1LC3-I↓, BECN1↓, EDN1, TP53↓, SQSTM1, p-TP53↓, p-RB1, NO↓, PUMA↓	autophagosome↓	[14]
		Ovarian cancer	ovary	PEO1, BG-1, SKOV-3R	1 μM	MAP1LC3-II, MAP1LC3-I, IFI27, PARP1, ESR1↓	autophagy (not alone)	[47, 48]

		Uterine endometrial carcinoma	uterus	Ishikawa and KLE cells	250 nM	MAP1LC3-II, MAP1LC3-I, BECN1, MYC, SQSTM1 ↓ , **Glutaminase**	autophagosome	[49]
	resver atrol	Breast cancer	breast	MCF-7, MCF10a, MDA-MB-231	100 μM	MAP1LC3-II ↓ , MAP1LC3-II/ MAP1LC3-I ↓	RAP-induced autophagy ↓	[50]
		Diabetic cardiac function l	heart	H9c2	100 μM	estrogen-regulated genes, RICTOR ↓ , AKT1	NA	[51]
		Pituitary tumor	pituitary gland	GH3	25, 50 μM	MAP1LC3-II/ MAP1LC3-I, BECN1, BCL2 ↓	NA	[52]
	raloxif ene	Amyotrophic lateral sclerosis	motor neuron	TDP-25	10^{-9}, 10^{-8}, 10^{-7}, 10^{-6} M	MAP1LC3-II ↓ , SQSTM1 ↓	NA	[53]
		Breast cancer	breast	MCF-7	10 μM	MAP1LC3-II, BECN1, ATG12–ATG5 conjugates	autophagic flux, autophagic vacuole	[54]
	TAM	Breast cancer	breast	MCF-7, MCF-7 tamR, LCC2, MDA-MB-231	1, 3, 5 μM	MAP1LC3-II/ MAP1LC3-I, BCL2, SQSTM1, BECN1, MAPK1-MAPK3, BAX-ATG12–ATG5	autophagic vacuole, autophagosome s	[56, 46, 5-58]
		Glioblastoma	brain	U87, X1016, JX6	9, 12 μM	MAP1LC3-II, MAP1LC3-I, ATG5 ↓	autophagic vacuole formation	[59]
		Toxoplasmosis	breast	MCF-7	5, 10 μM	MAP1LC3-II, MAP1LC3-I	NA	[60]
**ESR1 antagonist**	MPP	Arterial calcification	arteries	VSMCs	100 nM	MAP1LC3-II/ MAP1LC3-I ↓ , MAP1LC3-II ↓	NA	[43]
**ESR2 agonist**	DPN	Breast cancer	breast	MDA-MB-231	100 nM	MAP1LC3-II, ESR2, ATG5, ATG16, BECN1, UVRAG, **CLDN6**	autophagic vacuole	[28]
		Hodgkin lymphoma	lymph	L-428, KM-H2, L-540, HDLM-2	10 nM	ESR2, TP53, ULK1, DRAM2, ATG9, LC3-II, SQSTM1 ↓	autophagosome	[72]
		Osteosarcoma	bone	human osteosarcoma U2-OS	100 μM	MAP1LC3-II, MAP1LC3-I, ESR2, p-MTOR ↓ , SQSTM1 ↓	autophagosome	[61]
	L17, WAY-20070	Breast cancer	breast	MCF-7/LCC1, MCF-7/LCC9, MCF-7CL	10 nM	MAP1LC3-II/ MAP1LC3-I, BCL2 ↓ , ESR1 ↓	NA	[62]
**ESR2 antagonist**	PHTP P	Arterial calcification	arteries	VSMCs	100 nM	MAP1LC3-II/ MAP1LC3-I ↓ , MAP1LC3-II ↓	NA	[43]
**GPER1 agonist**	2-ME	Osteosarcoma	bone	MG63	10 μM	MAP1LC3-II/ MAP1LC3-I	NA	[63]
		Systemic sclerosis	skin	fibroblasts, HUVECs	5, 10, 25 mM for fibrobl asts; 0.5, 2.5 mM for HUVE Cs	MAP1LC3-II ↓ , MAP1LC3-I ↓ , SQSTM1	autophagosome/ autolysosome	[64]
	DHEA	atherosclerosis	cardiovascular	human aortic endothelial cells	0.1, 1 μM	MAP1LC3-II/MAP1LC3-I, SQSTM1 ↓	fusion of autophagosome and lysosome	[65]
		breast cancer	breast	MCF-7		PPARG, PTEN, AKT-MTOR pathway ↓ , p-BCL2, BECN1, MAP1LC3	autophagosome	[66]

		Tuberculosis	lung	Mtb-infected macrophages	0.1, 1 μM	RACK1, anti-glucocorticoid, **IL1B, IL10, TNF**	autophagosome	[233]
	genistein	Breast cancer	breast	MCF-7, (CCD1059sK	100 μM	MAP1LC3/ MAP1LC3B, BAX-BCL2	autophagosome	[67]
	G1	Cardiac hypertrophy (angiotensin II-induced)	heart	Wistar rats	1000 nM	MAP1LC3-II/ MAP1LC3-I ↓ , p-AKT1, p-MTOR, RPS6KB1, GPER1, EIF4EBP1 ↓	NA	[13]
		Neuronal damage (glutamate-induced)	cortical neuron	Mice astrocyte	1 nM	MAP1LC3-II/ MAP1LC3-I ↓ , p-AKT1, p-PIK3CA, p-MTOR, BECN1 ↓ , SQSTM1	autophagosome ↓	[68]
**GPER1 antagonist**	G15	Cardiac hypertrophy (angiotensin II-induced + G1)	heart	Wistar rats	100 nM	MAP1LC3-II/ MAP1LC3-I, p-AKT1 ↓ , p-MTOR ↓ , EIF4EBP1, GPER1 ↓ , RPS6KB1 ↓	NA	[13]
		Neuronal damage (glutamate-induced+G1)	cortical neuron	Mice astrocyte	100 nM	MAP1LC3-II/ MAP1LC3-I, p-AKT1 ↓ , p-PIK3CA ↓ , p-MTOR ↓ , BECN1, SQSTM1 ↓	autophagosome	[68]
		Oral squamous cell carcinoma	mouth	SCC4, SCC9, HSC-3	0-20 μM	MAP1LC3B-II, MAP1LC3B-I, BCL2 ↓ , AKT1 ↓ , MAPK1 ↓ , GPER1 ↓	autophagosome	[42]

Abbreviations: 2-ME, 2-methoxyestradiol; 4-OH TAM, 4-hydroxytamoxifen; BAX, BCL2 associated X, apoptosis regulator; BCL2, BCL2 apoptosis regulator; DHEA, dehydroepiandrosterone; DPN, diarylpropionitrile; EIF4EBP1, eukaryotic translation initiation factor 4E binding protein 1; IL10, interleukin 10; IL1B, interleukin 1 beta; MPP, methy-piperidinopyrazole; MTOR, mechanistic target of rapamycin kinase; MYC, MYC proto-oncogene, bHLH transcription factor; PIK3CA, phosphatidylinositol-4,5-bisphosphate 3-kinase catalytic subunit alpha; PHTPP, 2-phenyl-3-(4-hydroxyphenyl)-5, 7-bis (trifluoromethyl)-pyrazolo [1,5-alpha] pyrimidine; RICTOR, RPTOR independent companion of MTOR complex 2; RPS6KB1, ribosomal protein S6 kinase B1; SERM, selective estrogen receptor modulator; T47D, human ductal carcinoma of the breast; TAM, tamoxifen; TNF, tumor necrosis factor; ↓ , ESR ligand-induced downregulation; no arrow, ESR ligand-induced upregulation; bold font, effector proteins associated with other reactions in cells, such as inflammation, proliferation, migration, apoptosis, and immunity.

Table S3. Autophagy-related proteins regulated by estrogen via microRNAs.

**Gene**	**microRNAs**	**Effect of MiRNAs on Autophagy**	**Tissue or Cell Line**	**Ref**	**Receptor**	**Effect of E2 on MiRNAs**	**Tissue or Cell Line**	**Ref**
**Regulation of autophagy induction**								
RB1CC1	*MIR10A*	Suppress	MEFs HEK293T	[163, 166]	ESR2	E2 upregulates *MIR10A-5p*	MCF-7	[167]
ULK1	*MIR20A*	Suppress	C2C12	[169]	ESR1	E2 upregulates *MIR20A*	MCF-7	[170]
MTOR	*MIR214*	Suppress	MCF-7/LCC9	[176]	ESR1	E2 inhibits the expression of microRNA-214	Uteri of ovariectomized mice	[178]
	Mirlet7g	Enhance	Mouse granulosa cells	[164]	ESR1	E2 downregulates *Mirlet7g*	MCF-7, T47D	[165, 172]
**Phagophore nucleation**								
BCL2	*MIR21*	Suppress	INS-1 832/13	[171]	ESR1	E2 downregulates *MIR21*	HepG2, MCF-7, Isolated human endometrial glandular epithelial cells	[172, 173]
HMGB1	*HOTAIR*	Suppress	HuH28, HuCCT1	[187]	GPER1	E2 induces *HOTAIR* by suppressing *MIR148A*	MDA-MB-231, BT549	[179]

BECN1	*MIR21*	Suppress	MEFs	[174]	ESR1	E2 downregulates *MIR21*	HepG2, MCF-7, Isolated human endometrial glandular epithelial cells	[172, 173]
	HOTAIR	Enhance	Ishikawa Human endometrial cancer cells 786-O, ACHN	[180, 189]	GPER1	E2 induces *HOTAIR* by suppressing *MIR148A*	MDA-MB-231, BT549	[179]
**Phagophore expansion**								
ATG3	*HOTAIR*	Enhance	Hepatocellular carcinoma	[181]	GPER1	E2 induces *HOTAIR* by suppressing *MIR148A*	MDA-MB-231, BT549	[179]
ATG7	*MIR17*	Suppress	T98G	[168]	ESR1	E2 upregulates *MIR17*	MCF-7	[170]
	HOTAIR	Enhance	Hepatocellular carcinoma, PANC-1, AsPC-1	[181, 184]	GPER1	E2 induces *HOTAIR* by suppressing *MIR148A*	MDA-MB-231, BT549	[179]
ATG12	*MIR21*	Suppress	MEFs	[174]	ESR1	E2 downregulates *MIR21*	HepG2, MCF-7, Isolated human endometrial glandular epithelial cells	[172, 173]
NPTX2	*HOTAIR*	Enhance	MN9D	[192]	GPER1	E2 induces *HOTAIR* by suppressing *MIR148A*	MDA-MB-231, BT549	[179]

Abbreviations: 786-O, human renal clear cell adenocarcinoma cell; ACHN, human renal cell adenocarcinoma cells; AsPC-1, human metastatic pancreatic adenocarcinoma cells; BT549, human ductal carcinoma of the breast; C2C12, mouse myoblast cell; HMGB1, high mobility group box 1; HEK293T, human embryonic kidney cell; HuCCT1, cholangiocarcinoma cells; HuH28, cholangiocarcinoma cells; INS-1 832/13, rat insulinoma cells; MCF-7/LCC9, anti-estrogen-resistant strains of human breast cancer cells; MEFs, mouse embryonic fibroblasts; MN9D, mouse midbrain dopaminergic neurons; NPTX2, neuronal pentraxin 2; PANC-1, human pancreatic cancer cell; RB1CC1, RB1 inducible coiled-coil 1 (an autophagy inducer protein).
